# MEK/ERK signaling is a critical regulator of high-risk human papillomavirus oncogene expression revealing therapeutic targets for HPV-induced tumors

**DOI:** 10.1371/journal.ppat.1009216

**Published:** 2021-01-22

**Authors:** Adrian J. Luna, Rosa T. Sterk, Anastacia M. Griego-Fisher, Joon-Yong Chung, Kiersten L. Berggren, Virginie Bondu, Pamela Barraza-Flores, Andrew T. Cowan, Gregory N. Gan, Emrullah Yilmaz, Hanbyoul Cho, Jae-Hoon Kim, Stephen M. Hewitt, Julie E. Bauman, Michelle A. Ozbun

**Affiliations:** 1 Department of Molecular Genetics & Microbiology, The University of New Mexico School of Medicine, Albuquerque, NM, United States of America; 2 Experimental Pathology Laboratory, Laboratory of Pathology, Center for Cancer Research, National Cancer Institute, National Institutes of Health, Bethesda, United States of America; 3 Department of Surgery, Division of Otolaryngology, The University of New Mexico School of Medicine, Albuquerque, United States of America; 4 The University of New Mexico Comprehensive Cancer Center, Albuquerque, United States of America; 5 Department of Radiation Oncology, The University of Kansas Medical Center, Kansas City, United States of America; 6 Department of Internal Medicine, Division of Hematology and Oncology, The University of New Mexico School of Medicine, Albuquerque, United States of America; 7 Department of Obstetrics and Gynecology, Gangnam Severance Hospital, Yonsei University College of Medicine, Seoul, South Korea; 8 Department of Medicine, The University of Arizona Cancer Center, Tucson, United States of America; University of Wisconsin Madison School of Medicine and Public Health, UNITED STATES

## Abstract

Intracellular pathogens have evolved to utilize normal cellular processes to complete their replicative cycles. Pathogens that interface with proliferative cell signaling pathways risk infections that can lead to cancers, but the factors that influence malignant outcomes are incompletely understood. Human papillomaviruses (HPVs) predominantly cause benign hyperplasia in stratifying epithelial tissues. However, a subset of carcinogenic or “high-risk” HPV (hr-HPV) genotypes are etiologically linked to nearly 5% of all human cancers. Progression of hr-HPV-induced lesions to malignancies is characterized by increased expression of the E6 and E7 oncogenes and the oncogenic functions of these viral proteins have been widely studied. Yet, the mechanisms that regulate hr-HPV oncogene transcription and suppress their expression in benign lesions remain poorly understood. Here, we demonstrate that EGFR/MEK/ERK signaling, influenced by epithelial contact inhibition and tissue differentiation cues, regulates hr-HPV oncogene expression. Using monolayer cells, epithelial organotypic tissue models, and neoplastic tissue biopsy materials, we show that cell-extrinsic activation of ERK overrides cellular control to promote HPV oncogene expression and the neoplastic phenotype. Our data suggest that HPVs are adapted to use the EGFR/MEK/ERK signaling pathway to regulate their productive replicative cycles. Mechanistic studies show that EGFR/MEK/ERK signaling influences AP-1 transcription factor activity and AP-1 factor knockdown reduces oncogene transcription. Furthermore, pharmacological inhibitors of EGFR, MEK, and ERK signaling quash HPV oncogene expression and the neoplastic phenotype, revealing a potential clinical strategy to suppress uncontrolled cell proliferation, reduce oncogene expression and treat HPV neoplasia.

## Author summary

Human papillomavirus (HPV) infections occur in differentiating squamous epithelium and induce hyperplasia during the viral replicative cycle. Although HPV oncogene expression is necessary to promote cellular proliferation for viral genome amplification in the middle epithelial layers, oncogene levels are thereafter suppressed to permit differentiation-induced late gene expression in the uppermost epithelial cells. Yet, the mechanisms responsible for controlling HPV oncogene expression are not well understood. Here, we demonstrate that EGFR/MEK/ERK signaling, which is subject to the normal cellular cues of contact inhibition and epithelial tissue differentiation, is a critical regulator of hr-HPV oncogene expression. We found that extrinsic activation of ERK overrides cellular control to promote oncogene expression and the neoplastic phenotype. Many epidemiologically defined risk factors activate the EGFR/MEK/ERK pathway, suggesting a common mechanism whereby they may promote HPV persistence and disease progression. Lastly, we show that HPV oncogene transcription and protein expression remain susceptible to MEK/ERK control in early neoplastic tissues and tumor cells and that targeted inhibition of MEK/ERK signaling might be exploited therapeutically for HPV-induced infections and tumors.

## Introduction

Over 200 human papillomavirus (HPV) genotypes are recognized and can be grouped as high-risk (hr) or low-risk based on their ability to cause epithelial lesions with a high or low risk of malignant progression [1]. Oncogenic HPVs, including hr-HPV types 16, 18, and 31, have well-documented roles in the development of ≥99% of cervical cancers [2]. Hr-HPVs also are implicated in the etiology of cancers at other anogenital sites, and a large subset of oropharyngeal squamous cell carcinomas (OP-SCC) [3–5]. In all, hr-HPVs are estimated to be responsible for ≈5% of cancers worldwide [3]. Genetic approaches demonstrate that maintained expression of the hr-HPV E6 and E7 oncoproteins is necessary for the transformed phenotype *in vitro* and *in vivo* [6–8].

The complete replicative cycles of HPVs are complex and dependent on the differentiation program of stratified epithelium [9]. HPV infection of the basal epithelial cells initiates persistence *via* extra-chromosomal (episomal) nuclear replication of the circular viral genome. In the basal and suprabasal epithelial cells, early viral gene transcription is initiated from an early promoter, and hr-HPV early proteins promote host cell proliferation (E5, E6, E7) and viral genome amplification (E1, E2, E4) in the middle epithelial layers [10–13]. The hr-HPV E5, E6, and E7 oncogenes each are required for a productive infection as demonstrated in three-dimensional (3D) organotypic epithelial tissue cultures [10,11,14]. In uninfected epithelium and during productive HPV infections (e.g., cervical low-grade intraepithelial lesions [LSIL]), the suprabasal cells initiate a differentiation program. As infected cells leave the middle epithelial layers, presumably after amplification of viral genomes, early viral gene expression is suppressed; thereafter, expression of the capsid viral genes leads to progeny virion assembly [9]. Although keratinocyte differentiation cues are well known to activate late viral gene transcription [15], their contribution to subduing HPV early gene expression has not been studied. Thus, despite the fact that hr-HPV oncoproteins can inhibit differentiation, it remains enigmatic how hr-HPV early transcription is suppressed to reduce oncoprotein expression and permit differentiation in the middle epithelial layers during the productive HPV replicative cycle.

In contrast to a productive HPV infection, cervical high-grade intraepithelial lesions (HSIL) and HPV-positive cancers exhibit sustained expression of hr-HPV oncogenes and loss of epithelial differentiation in the middle-to-upper epithelial cell layers [12,16–18]. Although HPV E2 protein can suppress the viral early promoter when hr-HPV genomes are integrated into host cells, it has not been shown to do so in cells with episomal genomes [19]. Thus, the driving force behind increased oncogene expression during neoplastic progression is poorly understood.

The expression and activities of the epidermal growth factor receptor (EGFR) in epithelium parallel the pattern of HPV oncogene expression in productive HPV infections and early neoplasia [16]. EGFR, a receptor tyrosine kinase (RTK), plays a well-recognized role in keratinocyte proliferation and epithelium development [20,21]. Dividing keratinocytes in the basal epithelial layer display the greatest expression and activity of EGFR (i.e., phosphorylated-EGFR forms) [22], which sustains proliferation [23]. During the program of epithelial differentiation, EGFR expression is downregulated [24,25], and inhibiting EGFR activity blocks proliferation and induces early terminal differentiation of epidermal keratinocytes [26,27]. However, EGFR expression often remains elevated in hyperproliferative skin diseases like psoriasis and SCCs, albeit inconsistently in HPV positive SCCs [24,25,28]. Additionally, EGFR signaling and the levels of phospho-extracellular signal-regulated kinase 1/2 (p-ERK1/2), a downstream effector of RTKs *via* the mitogen-activated protein kinase kinase (MEK), increase during cervical intraepithelial neoplasia (CIN) progression from LSIL to HSIL [29–31]. Nevertheless, a mechanistic link between EGFR-mediated ERK1/2 signaling and HPV oncogene expression has not been investigated.

A substantive body of research indicates a link and crosstalk between proliferative signaling by EGFR and hr-HPV oncogene expression. EGF treatment of SiHa cervical cancer-derived cells led to increased HPV16 E6/E7 mRNA expression and this was dependent upon the presence of AP-1 transcription factor (TF) binding sites in the viral long control region (LCR) [32], a noncoding regulatory segment located upstream of the early gene coding region. AP-1 TFs are comprised of either homodimers of Jun proteins (c-Jun, JunB, JunD) or heterodimers of Jun with the Fos proteins (c-Fos, FosB, Fra-1, Fra-2) that transactivate gene expression by binding to heptamer consensus (5’-TGA[C/G]TCA-3’) and related sequences [33]. AP-1 TFs are well-known to be downstream effectors of EGFR and MEK/ERK signaling (reviewed by [33]), and, importantly, AP-1 binding sites are highly conserved in the LCRs across HPV genotypes [34]. AP-1 factors c-Fos, c-Jun and JunB were shown to directly mediate LCR-mediated transcription of hr-HPV types 16, 18, and 31 [35–37]. Additionally, the expression and activity of AP-1 TFs correlate with HPV oncogene transcription and cervical cancer progression from LSIL to HSIL [38–41]. These observations imply, but do not explicitly show, that EGFR and MEK/ERK signaling could be directly involved in promoting HPV oncogene expression, in addition to stimulating cell proliferation, in productive HPV infections and during cancer progression.

Despite the apparent, albeit fragmented, link between EGFR and ERK signaling and the expression of hr-HPV oncogenes, it has not been determined whether EGFR/MEK/ERK signaling has a role in HPV oncogene expression in infected human keratinocytes that maintain episomal viral genomes and model persistent early neoplastic infections. To this end, we investigated the mechanistic interplay between EGFR/ERK signaling and hr-HPV oncogene expression in epithelial cells and tissues. Our results from clinical lesions, cell cultures, and tissue models show a crucial role for MEK/ERK signaling in regulating hr-HPV oncogene expression in an epithelial tissue context. Furthermore, our findings reveal the requirement for MEK/ERK activity as a vulnerability that might be specifically targeted with clinically approved inhibitors in HPV-induced infections and cancers.

## Results

### Cervical clinicopathologic characteristics, p-ERK1/2, and p16INK4 expression

**Fig 1:**
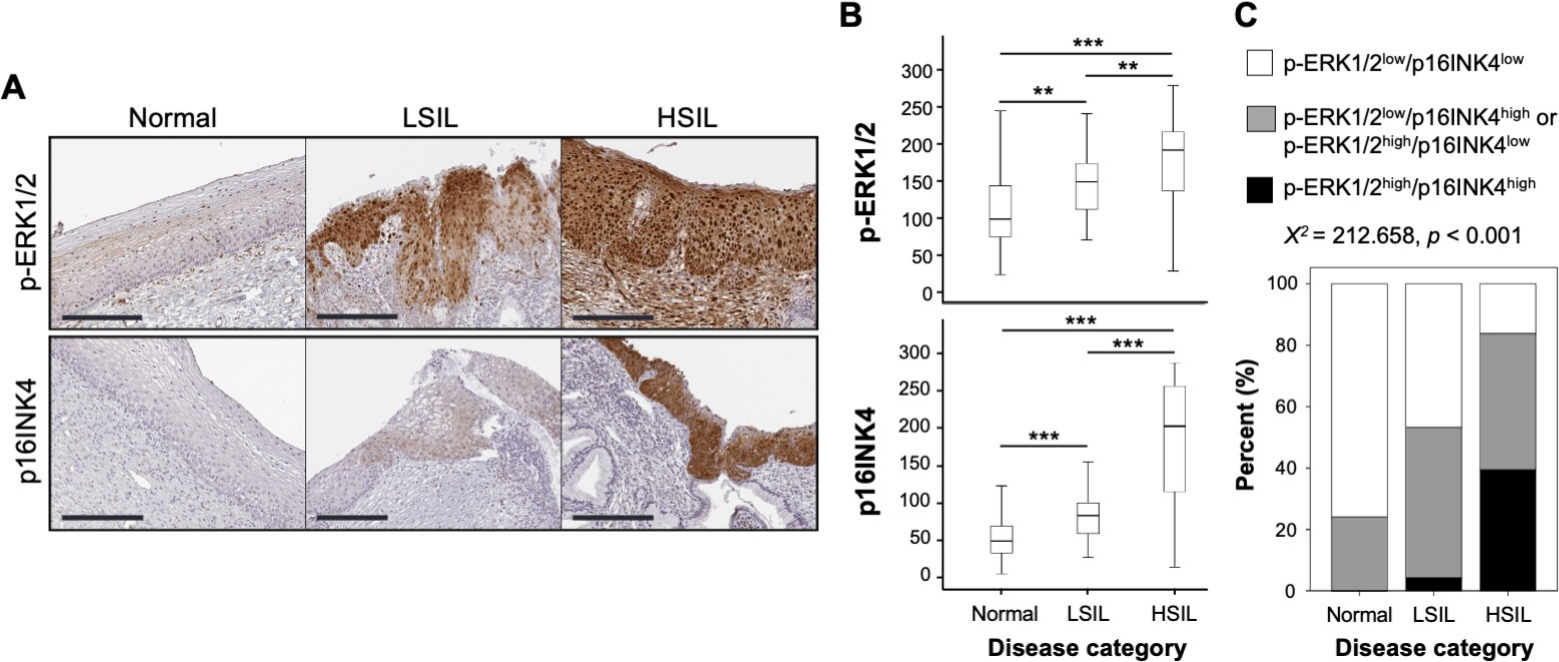
Correlation of p-ERK1/2 and p16INK4 expression in human cervical intraepithelial neoplasia specimens. (A) Representative IHC staining images of p16INK4 (p16) and p-ERK1/2 in normal, LSIL, and HSIL specimens. Bars equal 200 μm. (B) Box plot depiction of IHC scores; center tendency and variability. IHC scores for p-ERK1/2 and p16INK4 expression levels were significantly increased according to disease progression from normal to LSIL to HSIL (**p < 0.01 and ***p < 0.001 correspond to the non-parametric Kruskal-Wallis/Mann–Whitney U tests). (C) The combined expression of p-ERK1/2 and p16INK4 was compared among normal, LSIL, and HSIL samples (see S1 Table). The combined expression of p-ERK1/2^high^ and p16INK4^high^ was significantly increased in HSIL specimens compared to LSIL or normal samples (χ^2^ test; *p* < 0.001).

We previously showed that p-ERK1/2 overexpression is linked to clinical features of CIN progression from LSIL to HSIL, and is associated with poor prognosis and decreased disease-free and overall survival [29]. As the expression of hr-HPV E6 and E7 mRNAs show strikingly similar expression patterns and clinical associations with p-ERK1/2 signaling [9,29], we directly assessed the association between ERK activity and HPV oncogene expression during the transition of histologically normal cervical epithelia to LSIL and HSIL. In the same cohort of cervical tissues from patients with CIN lesions, we compared p-ERK1/2 IHC with that of p16INK4 (p16), an established surrogate marker of E7 expression [42]. In normal biopsies p-ERK1/2 expression was present in the lower epithelial layers as previously described in this cohort [29], whereas p16 expression was not readily observed (Fig 1). Cytoplasmic and nuclear p-ERK1/2 expression was increased in the lower layers and detection of p16 expression followed the same pattern in the CIN lesions (Fig 1A). p-ERK1/2 expression was progressively up-regulated from normal to LSIL to HSIL (Fig 1B and S1 Table). Likewise, p16 expression gradually increased according to the stages of cervical neoplastic progression, from normal tissues through LSIL and HSIL and strong expression of both p-ERK1/2 and p16 was often coincident throughout the HSIL tissues (Fig 1 and S1 Table). Furthermore, the combined expression of p-ERK1/2^high^/p16^high^ was significantly increased during the transition of normal to LSIL to HSIL (χ^2^ test; *p* < 0.001) (Fig 1C). These data show a strong association between ERK activity and HPV oncogene expression during HPV-induced CIN progression.

### EGF stimulates viral early gene transcription in HPV16(+) human keratinocytes

**Fig 2:**
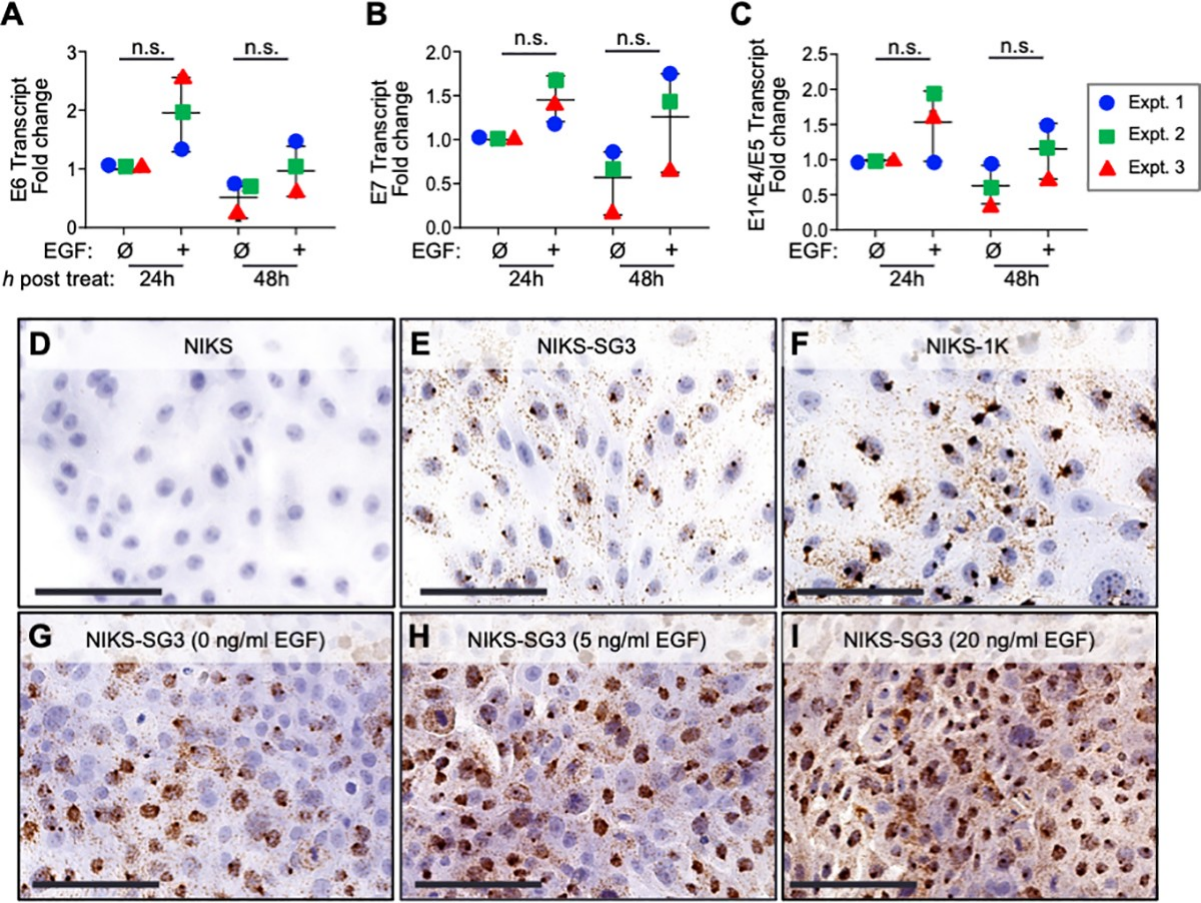
EGF stimulation results in increased viral transcription in the HPV16(+) NIKS-SG3 cell line. (A-C) Cells were incubated with (+) or without (Ø) 10 ng/ml EGF for the indicated times. Total RNA was subject to RT-qPCR for quantification of HPV early transcripts E6 (A), E7 (B) or E1^E4/E5 (C) (primer placement shown in S1 Fig). HPV mRNA levels were normalized to ribosomal protein 18s mRNA levels and are shown relative to the HPV mRNA levels in the 24h untreated cells for each experiment. The scatter plot shows the mean and range of the data from three independent experiments, wherein color-coded symbols show the data from independent experiments. The data were analyzed using 1-way ANOVA with Dunnett’s T3 multiple comparison test post hoc (not significant [n.s.]). (D-F) RNA ISH specific for hr-HPV E6/E7 RNAs on cells cultured with 10ng/ml EGF; (G-I) dose-effect of EGF on cells grown for 24h with or without added EGF (G-I). Bars equal 100 μm.

There are few, but conflicting reports on the effects of EGF stimulation on HPV oncogene transcription [32,43]. Thus, to investigate the cause-effect relationship between EGFR/ERK signaling and HPV oncogene expression we assessed the outcome of EGFR signaling in human keratinocytes that maintain episomal HPV16 genomes. Two independently-derived, HPV16 positive (+) cell lines, NIKS-SG3 and NIKS-1K, were developed from the near-diploid NIKS human keratinocyte cell line [44]. Both HPV16(+) cell lines initiate late virus life cycle stages when cultured as differentiating 3D-organotypic epithelial tissues, and thus model persistently infected, neoplastic SIL phenotypes [45,46]. To determine how EGF stimulation affected viral transcription in the proliferative context of episomally-replicating HPV16 genomes, subconfluent NIKS-SG3 cells were treated with 10 ng/ml EGF. HPV RNA levels were quantified by RT-qPCR targeting unspliced E6 and/or E7 mRNAs, as well as spliced E1^E4/E5 mRNAs. In each of three independent experiments with proliferating cells, EGF stimulation for 24h and 48h led to slight (≤2-fold) but consistently higher levels of HPV oncogene transcripts compared to untreated controls (Fig 2A–2C). Although the combined results of three independent experiments failed to reach statistical significance, the averaged oncogene mRNA levels were moderately higher in EGF-treated cells. Increased oncogene mRNA levels upon EGF stimulation were also observed using RNA *in situ* hybridization (RNA ISH) to detect polycistronic E6/E7-containing transcripts. Consistent with RT-qPCR data, EGF stimulation led to an increase in HPV16 E6/E7 RNA levels (Fig 2G–2I). The specificity of the RNA ISH was confirmed with controls: no positive RNA ISH signals were observed in the uninfected NIKS parental cells (Fig 2D), whereas the two HPV16(+) NIKS-derived cell lines were positive by RNA ISH (Fig 2E and 2F). Curiously, the time-course EGF stimulation experiments suggested that HPV early gene expression was negatively affected by increasing cell density. As the cells become more confluent from 24h to 48h, the HPV E6, E7, and E1^E4/E5 mRNA levels tended to decrease (Fig 2A–2C). These results indicate that EGF exposure has only a modest positive effect on HPV early gene transcription in proliferating cells with replicating episomal viral genomes. However, the findings also suggest that increasing cell density is a potential confounding factor in studying HPV oncogene expression and requires more directed investigation.

### Cell confluence and contact inhibition negatively regulate HPV oncoprotein expression

**Fig 3:**
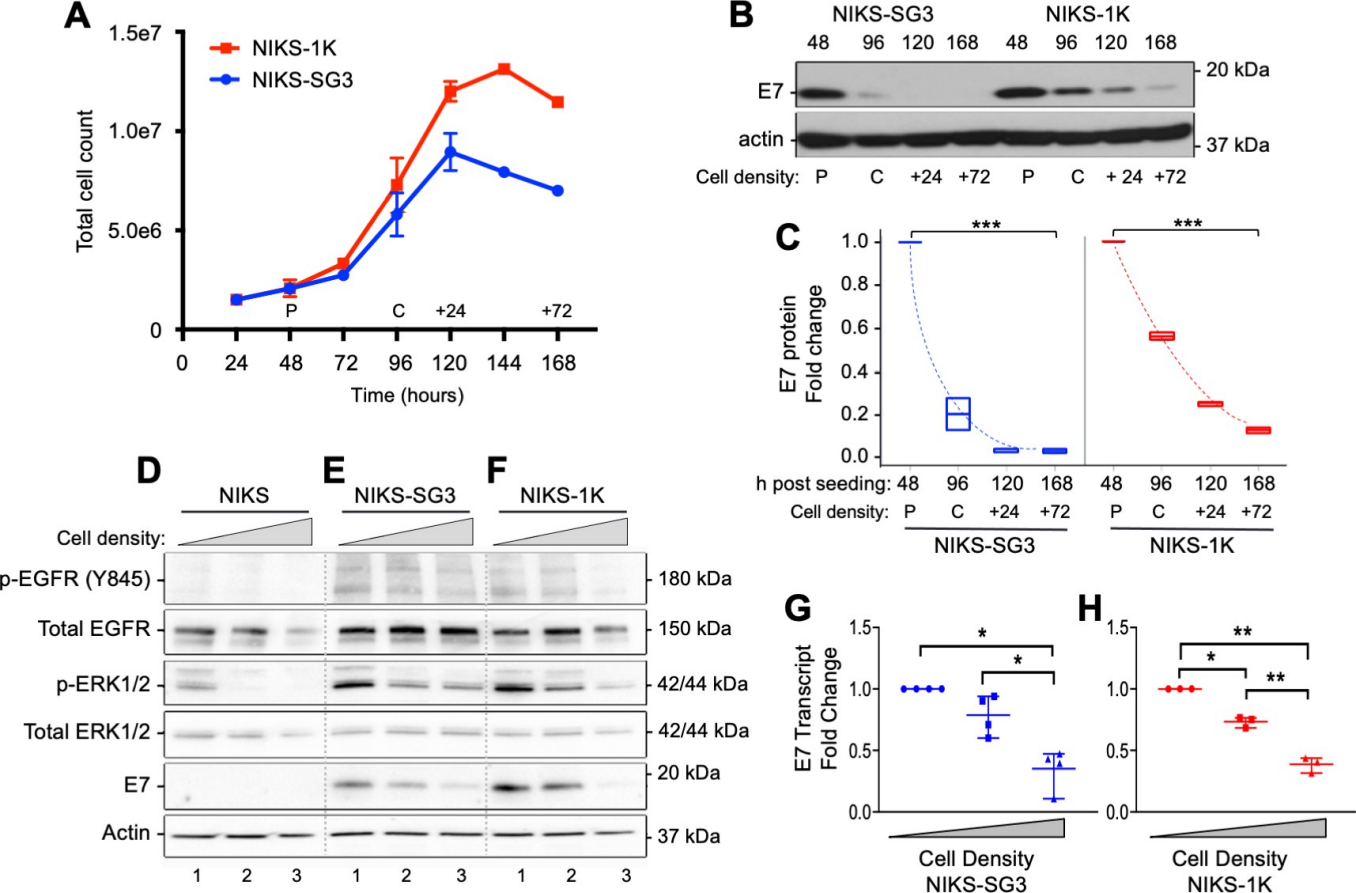
HPV oncoprotein expression is negatively regulated by increasing cell confluence and loss of EGFR/ERK signaling. (A) Growth curves constructed by seeding 1.5x10^6^ cells in 21cm^2^ plates and counting cells every 24h over 168h (n = 2; error bars = SD). Pre-confluent and confluent cell cultures (denoted as “P” and “C”, respectively) were defined by visual inspection of two biological replicates. (B) Cells were seeded as in (A) and proteins extracted when the cells were 70% (pre-) confluent (P), 100% confluent (C), 24h after reaching confluence (+24), and 72h after confluence (+72), corresponding to times indicated in (A). Protein lysates (25μg/lane) analyzed by SDS-PAGE and immunoblot (IB). (C) Densitometry analyses on IB data from two biological replicates as in (B). Box plots represent the median and range of the relative levels of HPV16 E7 protein for each cell density compared to the pre-confluent samples. Analyses included the random effects linear regression model with random slope, quadratic time (F = 521.11, p<0.0001 for time linear term and F = 226.02, p<0.0001 for quadratic time term), and interaction effect (F = 17.53, p = 0.0007 for the interaction term). (D-H) In parallel, plates of decreasing surface area (21cm^2^, 9.0cm^2^ and 4.6cm^2^) were seeded with 1.25x10^6^ cells, grown for 24h. (D-F) Protein lysates collected 24h post seeding, subjected to SDS-PAGE and IB. (G, H) Total RNA isolated 24h post seeding subjected to RT-qPCR targeting E7 mRNAs, normalized to ribosomal protein18s mRNA levels and lowest cell density sample. Scatter plots represent mean and range of the data, which were analyzed by 1-way ANOVA and Dunnett’s T3 multiple comparison test (*p<0.05, **p<0.01, ***p<0.001).

To determine the impact of cell confluence on viral gene expression in a controlled manner, we seeded replicate cultures of HPV16(+) NIKS-derived cell lines at equal cell densities and analyzed their growth and viral gene expression patterns in serum-containing medium lacking exogenous EGF. We found the NIKS-1K cell line grew past confluence and continued to proliferate before demonstrating contact inhibition of cell growth (Fig 3A). This is consistent with NIKS-1K data reported by Isaacson Wechsler *et al*., who also showed NIKS-1K cells give rise to an HSIL phenotype when grown as organotypic epithelial tissues [46]. In contrast, the NIKS-SG3 cell line, which demonstrates an LSIL phenotype in organotypic epithelial tissue culture [45], became contact inhibited and ceased dividing ≈24 h earlier than NIKS-1K cells (Fig 3A). We assessed the expression of the E7 oncoprotein in these cells harvested at increasing cell densities. Whereas the E7 oncoprotein was detectable in preconfluent cultures of both HPV16(+) NIKS cell lines, E7 protein levels decreased significantly as the cell cultures became contact inhibited (Fig 3B and 3C). Interestingly, NIKS-SG3 cells, whose proliferation plateau was reached more quickly than NIKS-1K cells, lost E7 expression at a significantly more rapid rate, whereas slower loss of E7 expression post confluence correlated with reduced contact inhibition in NIKS-1K cells (Fig 3A–3C). These results raised the possibility that altered cell signaling linked to cellular contact inhibition is important for the regulation of HPV oncoprotein expression.

### HPV oncogene expression and EGFR/MEK/ERK activity diminish as cells grow to higher densities

There are well-established roles for cell confluency and contact inhibition in suppressing RTK signaling in monolayer cell cultures (reviewed in [47]). Curto *et al*. showed global tyrosine phosphorylation and EGFR activity is reduced in non-tumorigenic cell lines as cell densities increase [48]. Thus, we determined the effects of increasing cell density on cellular tyrosine phosphorylation and EGFR activity in HPV(+) cell lines that maintain episomal viral genomes of hr-HPV genotypes 16, 18, or 31 derived by both transfection and from clinical CIN lesions. To rule out time in culture as a variable, we seeded equal numbers of cells into dishes with decreasing surface areas in medium lacking exogenous EGF (S2A Fig). Similar to the results reported by Curto and coworkers, tyrosine phosphorylation decreased with increasing cell density in each of the HPV(+) cell lines after 24h in culture (S2B Fig).

Concomitant with decreased global tyrosine phosphorylation and increasing cell density, EGFR/MEK/ERK signaling, measured by p-EGFR and p-ERK1/2, was suppressed in all cells examined (Figs 3D–3F and S2D). Although total levels of EGFR and ERK1/2 were unaltered or slightly diminished by increasing cell density of NIKS and the two HPV16(+) NIKS cell lines, both p-EGFR (Y845) and p-ERK1/2 were suppressed (Fig 3D–3F). Similar results were seen in the CIN 1-derived HPV16(+) W12-E cell line, the HPV18(+) NIKS-H18 cell line, and the CIN 1-derived HPV31(+) CIN-612 9E cell line, (S2D Fig).

The suppression of EGFR/MEK/ERK signaling with increasing cell density correlated with reduced HPV oncoprotein levels and their mRNA transcripts. Increasing cell density led to reduced E7 protein levels in HPV16(+) and HPV18(+) cells (Figs 3E, 3F, S2D and S2E). Although challenging to detect in most HPV infected cells, E6 protein levels were also reduced following increasing cell density (S2D and S2E Fig). Lacking antibodies that detect HPV31 E6 and E7 proteins, we were unable to correlate the reduced EGFR activity with HPV31 oncoprotein expression (S2C Fig). Transcriptional suppression of HPV E6 and E7 mRNAs closely followed the cell-regulated decreases in EGFR/MEK/ERK signaling in each HPV(+) cell line tested (Figs 3G, 3H, S2F and S2G). These data suggest that HPV oncogene transcription is closely tied to EGFR/MEK/ERK signaling.

Notably, direct comparisons revealed that HPV16(+) NIKS cells consistently showed moderately higher p-EGFR and p-ERK1/2 levels than parental, uninfected NIKS cells (Fig 3D–3F), but proliferating cells demonstrated similar plasma membrane levels of EGFR (S2C Fig). These data suggest that persistent HPV infection can augment EGFR/ERK signaling without substantially altering total EGFR expression. Yet importantly, these non-transformed HPV(+) cells remained responsive to the growth inhibitory cues of cell contact inhibition, suggesting that HPV oncoproteins do not dominate EGFR/MEK/ERK activities in these cells. Rather, these data indicate that EGFR/MEK/ERK signaling governs control over hr-HPV oncogene expression in HPV-infected cells with early neoplastic phenotypes.

### Extrinsic EGFR stimulation overrides contact inhibition, MEK/ERK signaling suppression and HPV oncogene expression in high-density cell populations

**Fig 4:**
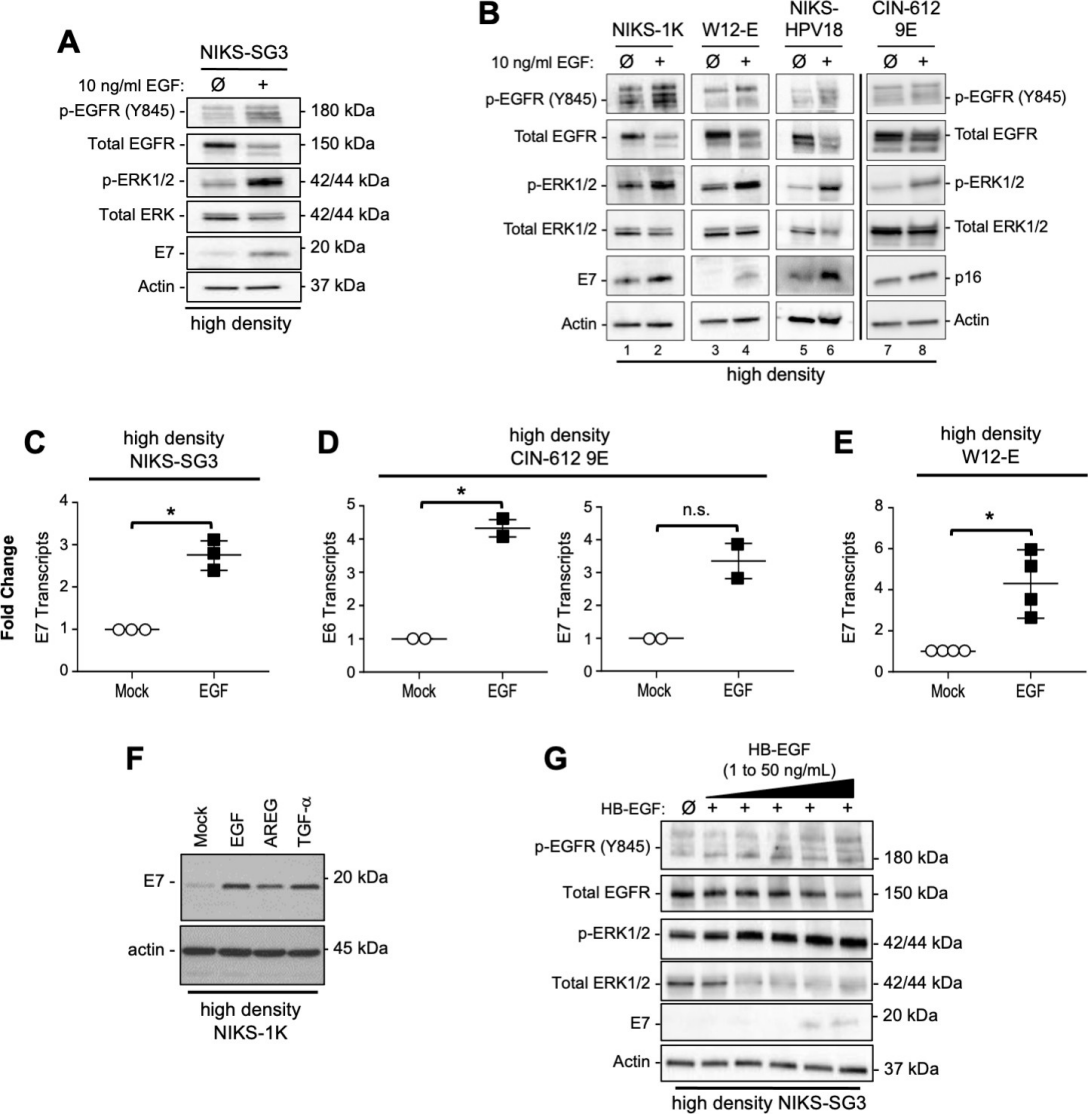
EGFR ligands rescue cell density-dependent suppression of EGFR/MEK/ERK signaling and HPV oncogene expression in HPV16(+) and HPV31(+) cells. (A-E) Cells were seeded at high cell density (1.3 x 10^5^ cells/cm) and allowed to attach overnight; cells were then treated with or without 10 ng/ml EGF for 24h before harvest. (A,B) Proteins (25 μg) subject to SDS-PAGE and IB. (B) In lanes 1–6, E7 proteins were detected; lacking antibodies to HPV31 E7 protein, p16 was detected as an E7 surrogate (lanes 7–8). (C-E) Total mRNAs extracted from replicate cultures were subjected to RT-qPCR targeting HPV oncogene mRNA; cDNA levels were normalized to ribosomal protein 18s mRNA levels and unstimulated sample. Scatterplots represent the mean and range of the data as analyzed by Welch’s t-test (*p<0.05). (F) High density cells were treated with 10 ng/ml each of EGF, AREG or TGF-α for 48h; proteins were subject to SDS-PAGE and IB. (G) Cells seeded at a high cell density were treated 24h with increasing concentrations of HB-EGF; proteins were subject to SDS-PAGE and IB.

Stimulation with EGFR ligands is reported to restore EGFR signaling in contact inhibited epithelial cells [49–51]. Thus, we seeded cells at high density and cultured overnight. One set of cells was untreated and the second was exposed to 10 ng/mL EGF for 24h. As compared to unstimulated cells in the confluent state, EGF stimulation re-activated EGFR signaling along with increased E7 oncoprotein expression in each of the cell lines (Fig 4). Lacking specific antibodies that can detect HPV31 E7, we detected p16 as a surrogate of E7 protein induction in CIN-612 9E cells (Fig 4B, lanes 7–8). In each HPV(+) cell line, increases in oncoprotein expression were mirrored by increased oncogene mRNA levels (Fig 4C–4E). The EGFR ligands amphiregulin (AREG), transforming growth factor alpha (TGF-α), and heparin-binding EGF (HB-EGF) also stimulated E7 protein expression in confluent HPV(+) cells (Fig 4F and 4G). Thus, extrinsic EGFR stimulation rescues contact inhibition-mediated EGFR/MEK/ERK suppression of signaling to stimulate HPV oncogene expression. In total, these results indicate that active EGFR signaling promotes HPV oncogene expression independent of cell density, hr-HPV genotype, or cell line background.

### MEK/ERK signaling is the dominant pathway influencing HPV oncogene expression

**Fig 5:**
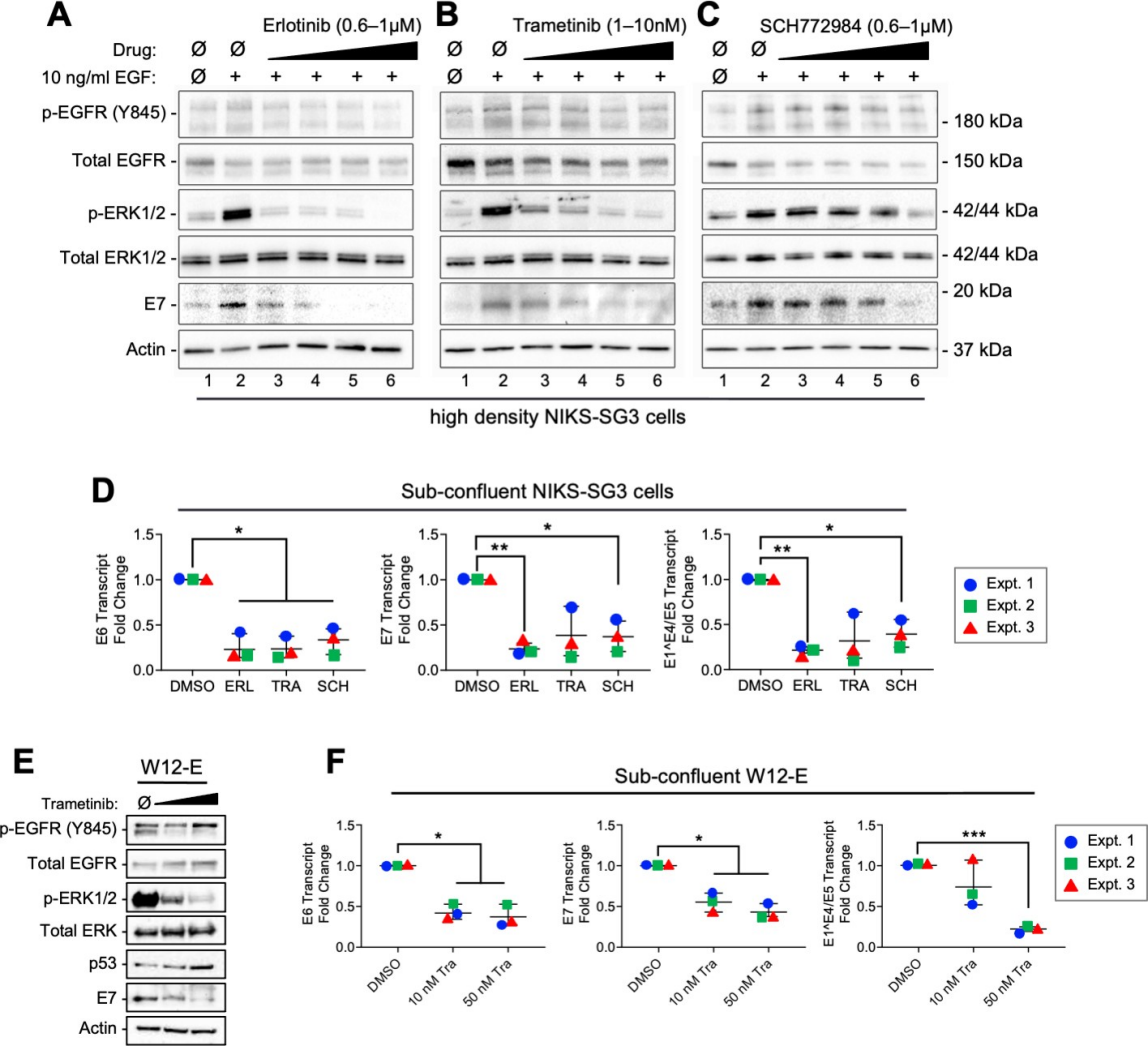
HPV oncogene expression is dependent upon EGFR/MEK/ERK activity in monolayer cell cultures. (A-C) Contact inhibited SG3 cells were grown in the presence of increasing concentrations of erlotinib, trametinib or SCH772984 for 8h prior to EGF stimulation. Protein lysates were collected 14h after EGF stimulation and subject to SDS-PAGE and IB. (D) Cells seeded at low density (2.3 x10^4^ cells/cm) were treated with 1μM erlotinib, 10nM trametinib, or 1μM SCH772984 for 24 hours. Total RNAs extracted from replicate cultures were subjected to RT-qPCR for HPV transcripts and levels were normalized to ribosomal protein 18s mRNA levels. Vehicle treated (DMSO) was set to one in each experiment and the data were analyzed by 1-way ANOVA and Dunnett’s T3 multiple comparison test (*p<0.05 or **p<0.01). (E-F) W12-E cells were seeded at low density (3.12 x10^4^ cells/cm) treated with 10 nM or 50 nM trametinib for 24 hours. (E) Protein (50 μg) was subject to SDS-PAGE and IB. (F). Total RNAs extracted from replicate cultures were subjected to RT-qPCR for HPV transcript and normalized to ribosomal protein 18s mRNA levels. Vehicle treated (DMSO) was set to one in each experiment and the data was analyzed by 1-way ANOVA with Dunnett’s T3 multiple comparison post hoc test (*p<0.05, **p<0.01, ***p<0.001).

EGFR signaling can activate the MEK/ERK, PI3K/AKT, PKC, and p38 MAPK pathways, which promote cellular proliferation, survival, and/or migration. To determine whether the regulation of oncogene expression by EGF stimulation of HPV(+) cells was primarily dependent upon MEK and ERK signaling downstream of EGFR, we performed a dose-escalation experiment using small molecule drug inhibitors. EGFR was targeted with erlotinib, a reversible, ATP-competitive inhibitor of EGFR tyrosine phosphorylation [52]. MEK1/2, the ERK1/2 kinase, was selectively inhibited with trametinib [53], and SCH772984 was used as a selective inhibitor that prevents ERK1/2 phosphorylation and activity [54]. High density NIKS-SG3 cells were grown in the presence of increasing drug doses for 8h prior to EGF stimulation. Similar to data shown in Fig 4, when compared to unstimulated cells, EGF treatment strongly induced p-EGFR, p-ERK/1/2 and E7 expression (Fig 5A–5C, compare lanes 1 and 2). We found that each drug inhibited its target in the presence of EGF stimulation, converging in dose-dependent p-ERK1/2 suppression (Fig 5A–5C, lanes 3–6). In each case suppression of ERK1/2 phosphorylation closely mirrored the dose-dependent decrease in E7 protein levels (Fig 5A–5C, lanes 3–6), and each drug treatment also resulted in strong suppression of HPV oncogene transcription (Fig 5D). Our findings that separate inhibitors targeting EGFR, MEK or ERK signaling led to a dose-dependent suppression of p-ERK1/2 with concomitant decreases in HPV oncogene expression minimizes the possibility that off-target drug effects contributed to oncogene regulation. Similarly, W12-E cells treated with the MEK inhibitor, trametinib responded with suppressed p-ERK1/2 and E7 levels; p53 levels increased with trametinib treatment suggesting that E6 protein expression was reduced (Fig 5E). Likewise, HPV oncogene transcription was suppressed in W12-E cells treated with the MEK inhibitor (Fig 5F). Together, these results indicate that MEK/ERK signaling downstream of EGFR is a crucial node regulating hr-HPV oncogene transcription and oncoprotein expression.

Due to the frequent activation of the PI3K/AKT pathway in HPV positive cancers [55–57], we evaluated whether EGF stimulation of this pathway contributed to E7 oncoprotein expression in cells maintaining episomal HPV genomes. Briefly, W12-E cells were grown at high density, treated for 48h with increasing doses of BYL719, a selective p110α inhibitor, then stimulated with EGF. Whereas the highest concentration of BYL719 inhibited phosphorylation of AKT, this did not hinder the rescue of viral oncoprotein expression (S3 Fig, lane 6). These data suggest that within the context of contact inhibition, the PI3K/AKT pathway does not play a substantive role in regulating hr-HPV oncogene expression downstream of EGFR.

### MEK/ERK signaling promotes AP-1 transcription factor activity to activate HPV oncogene transcription

**Fig 6:**
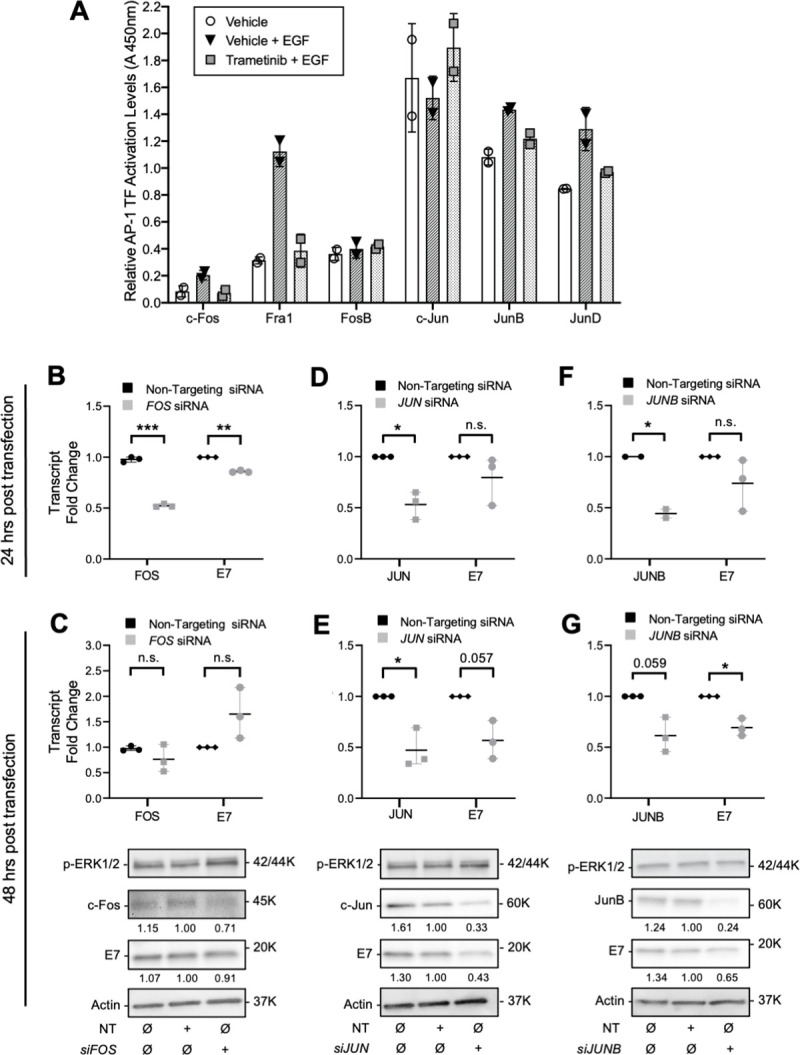
AP-1 transcription factors regulate HPV oncogene expression. (A) W12-E cells were seeded at high density and grown for 8h in the presence of DMSO or 50 nM trametinib prior to EGF stimulation. Nuclear extracts were collected 14h after EGF stimulation and assayed for the levels of each active AP-1 factor. (B-G) W12-E cells were serum starved for 4h prior to transfection with commercially available siRNA pools targeting *FOS*, *JUN* or *JUNB* mRNAs or non-targeting (NT) control siRNAs; these mRNAs encode the proteins c-Fos, c-Jun, or JunB, respectively. (B,D,F) Total mRNAs were extracted at 24h post transfection and subjected to RT-qPCR to quantify the AP-1 mRNAs and HPV E7 oncogene mRNAs. cDNA levels were normalized to those of ribosomal protein 18s and the values for NT siRNA transfected samples were set to one in each experiment. (C,E,G) At 24h post transfection, medium was replaced with serum-containing medium prior to harvesting for RNA and protein analyses at 48 post transfection Scatterplots represent the mean and range of the RT-qPCR data, which were analyzed by an unpaired Welch’s t-test (*p<0.05, **p<0.01, ***p<0.001). Protein lysates collected 48h post transfection were subjected to SDS-PAGE, IB and densitometry. Values directly below each lane represent the protein levels normalized to the NT siRNA transfected samples in each experiment.

As AP-1 TFs are activated by serum and growth factor stimulation *via* ERK1/2, among other kinases [33,58,59], we assessed the activity of AP-1 TFs in nuclear extracts from W12-E cells. Thus, we determined which AP-1 TFs were activated under conditions that enhance and suppress HPV oncogene transcription. Levels of active AP-1 TFs were compared among cells grown at high-density to suppress p-ERK1/2 activity and oncogene transcription, high-density cells stimulated for 14 h with EGF to increase both, and cells stimulated with EGF following treatment for 8h in the presence of MEK inhibitor, trametinib, to specifically inhibit p-ERK1/2 signaling (Fig 6A). We found that activity of c-Fos, Fra-1, JunB, and JunD were specifically increased with p-ERK1/2 signaling, whereas the activity of FosB and c-Jun were not appreciably affected by p-ERK1/2 stimulation. Previous studies showed c-Fos, c-Jun and JunB directly mediated LCR-mediated transcription of hr-HPVs [35–37,60], whereas JunD and Fra-1 appeared to negatively impact HPV transcription or were not involved [38,39,61,62]. We therefore assessed whether knockdown of c-Fos and JunB, whose activity was activated by p-ERK1/2 signaling, and c-Jun, which was not p-ERK1/2 responsive, influenced the expression of HPV oncogenes in cells maintaining episomal HPV genomes. HPV16(+) W12-E cells were serum starved to promote transfection and minimize ERK1/2 signaling. Cells were assayed at either 24h post transfection or at 48h post transfection following 24h of stimulation with medium containing 10% FBS to promote p-ERK1/2 activity. At 24h post transfection, cells receiving siRNAs to c-Fos-, c-Jun-, or JunB-encoding mRNAs showed an average AP-1 target mRNA knockdown of ≈50% compared to those transfected with non-targeting siRNAs (Fig 6B, 6D and 6F). At 24h post transfection E7 mRNAs were diminished significantly in cells with reduced c-Fos-encoding mRNAs (Fig 6B). However, both c-Fos- and E7-encoding mRNA levels rebounded and E7 protein levels were not appreciably altered in the presence of p-ERK1/2 at 48 h post transfection, following 24 h of serum stimulation (Fig 6C). Knockdown of c-Jun- and JunB-encoding mRNAs and their proteins at 48h post transfection in the presence of p-ERK1/2 were each accompanied with reduced E7 transcript and protein levels (Fig 6E–6G). Although it is unclear why the c-Fos-encoding mRNA knockdown was short lived upon serum stimulation and p-ERK1/2 activation, oncogene mRNA levels followed those of the AP-1 factors. These data are consistent with the idea that AP-1 TFs are needed for the expression of the HPV oncogenes and that c-Fos and JunB control oncogene transcription downstream of MEK/ERK signaling.

### EGFR/MEK/ERK signaling regulates HPV oncogene expression in organotypic epithelial tissues

**Fig 7:**
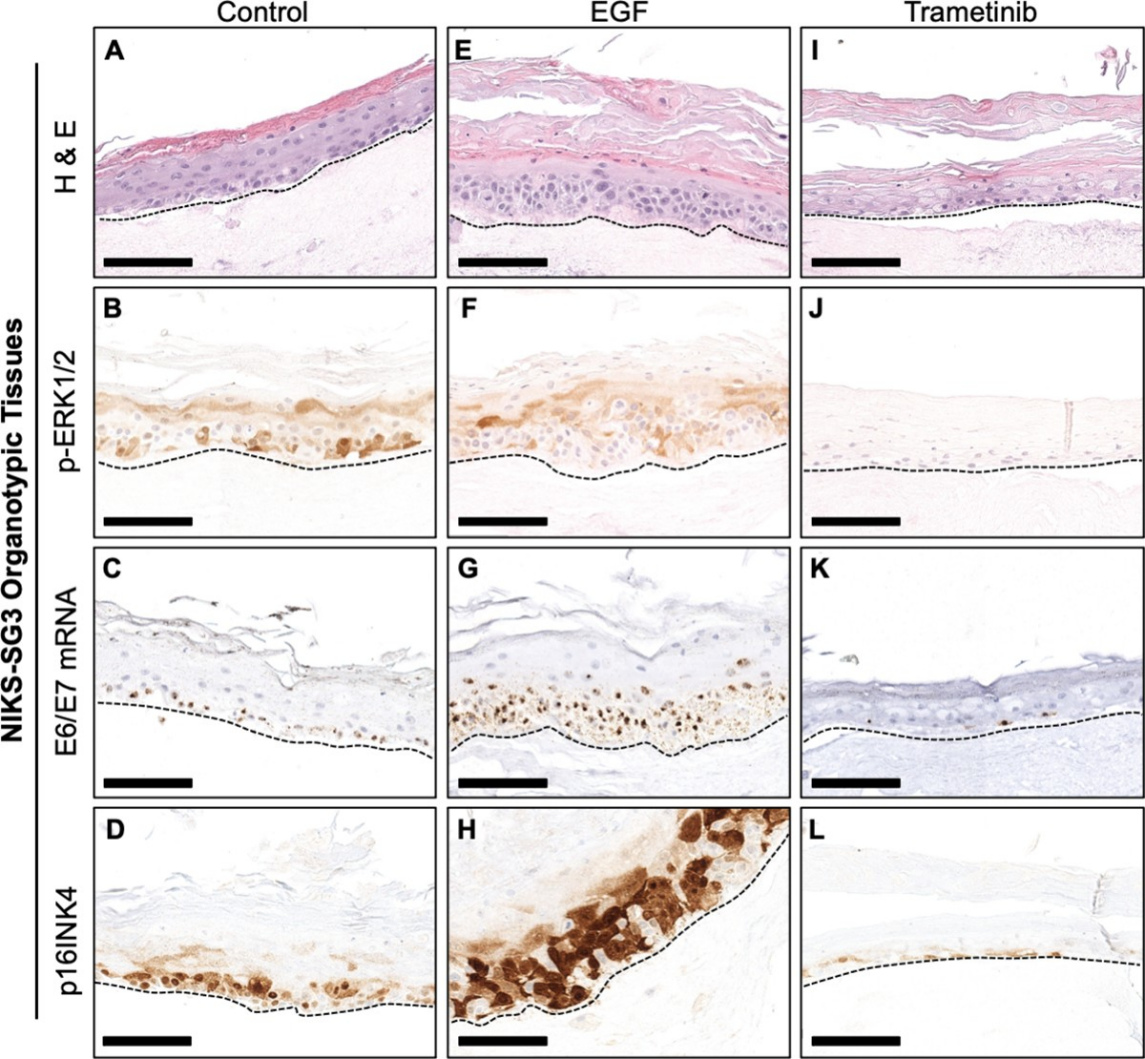
EGFR and MEK signaling regulates HPV oncogene expression in 3D-organotypic epithelial tissue models. Representative images of organotypic epithelial tissues from NIKS-SG3 cells grown at the air-liquid interface for 8 days prior to treatment with 10 ng/ml EGF or 10 nM trametinib every second day. FFPE tissues harvested on day 14 were sectioned and stained: (A, E, I) H&E; (B, F, J) IHC for p-ERK1/2; (C, G, K) RNA ISH (brown); (D, H, L) IHC for p16. Broken lines underscore the basal cell layer.

**Fig 8:**
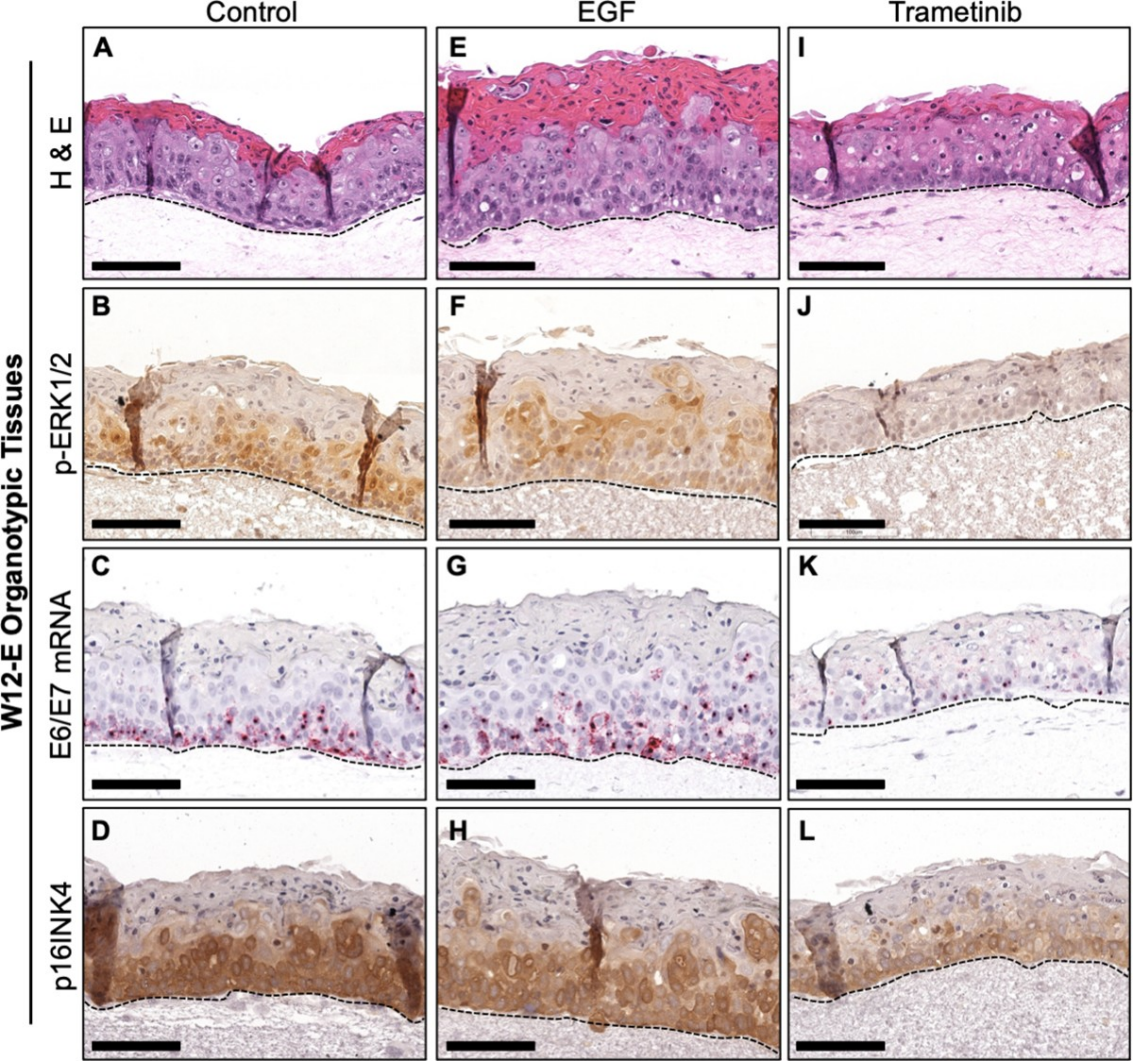
EGFR and MEK signaling regulates HPV oncogene expression in 3D-organotypic epithelial tissue models. Representative images of organotypic epithelial tissues from W12-E cells grown at the air-liquid interface for 8 days prior to treatment with 10 ng/ml EGF or 10 nM trametinib every second day. FFPE tissues harvested on day 14 were sectioned and stained: (A, E, I) H&E; (B, F, J) IHC for p-ERK1/2; (C, G, K) RNA ISH (red); (D, H, L) IHC for p16. Broken lines underscore the basal cell layer.

To assess whether EGFR/MEK/ERK activation was relevant for the regulation of HPV oncogene expression during the differentiation-dependent viral replicative cycle, we examined organotypic epithelial tissues of NIKS-SG3 and W12-E cells (Figs 7 and 8). Organotypic NIKS-SG3 and W12-E tissues grown in the absence of exogenous EGF demonstrated stratification as seen by an organized basal layer and an uppermost cornified layer, (Figs 7A and 8A). Nuclear p-ERK1/2 and E6/E7 mRNA were detected predominantly in the basal layer cells in the NIKS-SG3 tissues which demonstrate an LSIL phenotype (Fig 7B and 7C). W12-E organotypic tissues exhibited a dysplastic phenotype with increased nuclear p-ERK1/2 staining and E6/E7 mRNA detection higher throughout the tissue (Fig 8B and 8C). As E6 and E7 antibodies fail to detect the oncoproteins in FFPE sections, we stained the tissues for the E7 surrogate p16INK4 (p16) [42]. In agreement with the E6/E7 RNA ISH results in untreated epithelial tissues, p16 staining was strongest in the basal layer with lightly positive scattered cells in the intermediate epithelial layers of the NIKS-SG3 tissue and strongly positive cells in the W12-E intermediate tissue layers (Figs 7D and 8D). This concordance of E6/E7 RNA ISH and p16 localization and expression are consistent with data from HPV16-infected lesions [63]. In contrast, NIKS-SG3 and W12-E epithelial tissues stimulated with EGF for the last 6 days of growth demonstrated a differentiated but hyperplastic epithelium, and concomitantly, increased levels of p-ERK1/2, E6/E7 mRNAs, and p16 were observed throughout the basal and suprabasal cells (Figs 7E–7H and 8E–8H). Inhibition of MEK signaling with trametinib preserved epithelial differentiation but strongly suppressed p-ERK1/2 in both NIKS-SG3 and W12-E tissues (Figs 7I–7J and 8I–8J). In parallel, E6/E7 mRNAs and p16 were profoundly reduced (Figs 7K–7L and 8K–8L). In total, the monolayer and 3D-epithelial tissue data indicate that EGFR/MEK/ERK signaling is a crucial regulator of epithelial proliferation and of hr-HPV oncogene expression from episomally-replicating viral genomes during a productive hr-HPV infection in epithelium.

### EGFR/MEK/ERK regulates HPV oncogene expression in HPV(+) cancer cell lines cultured in monolayer and as organotypic tissues

**Fig 9:**
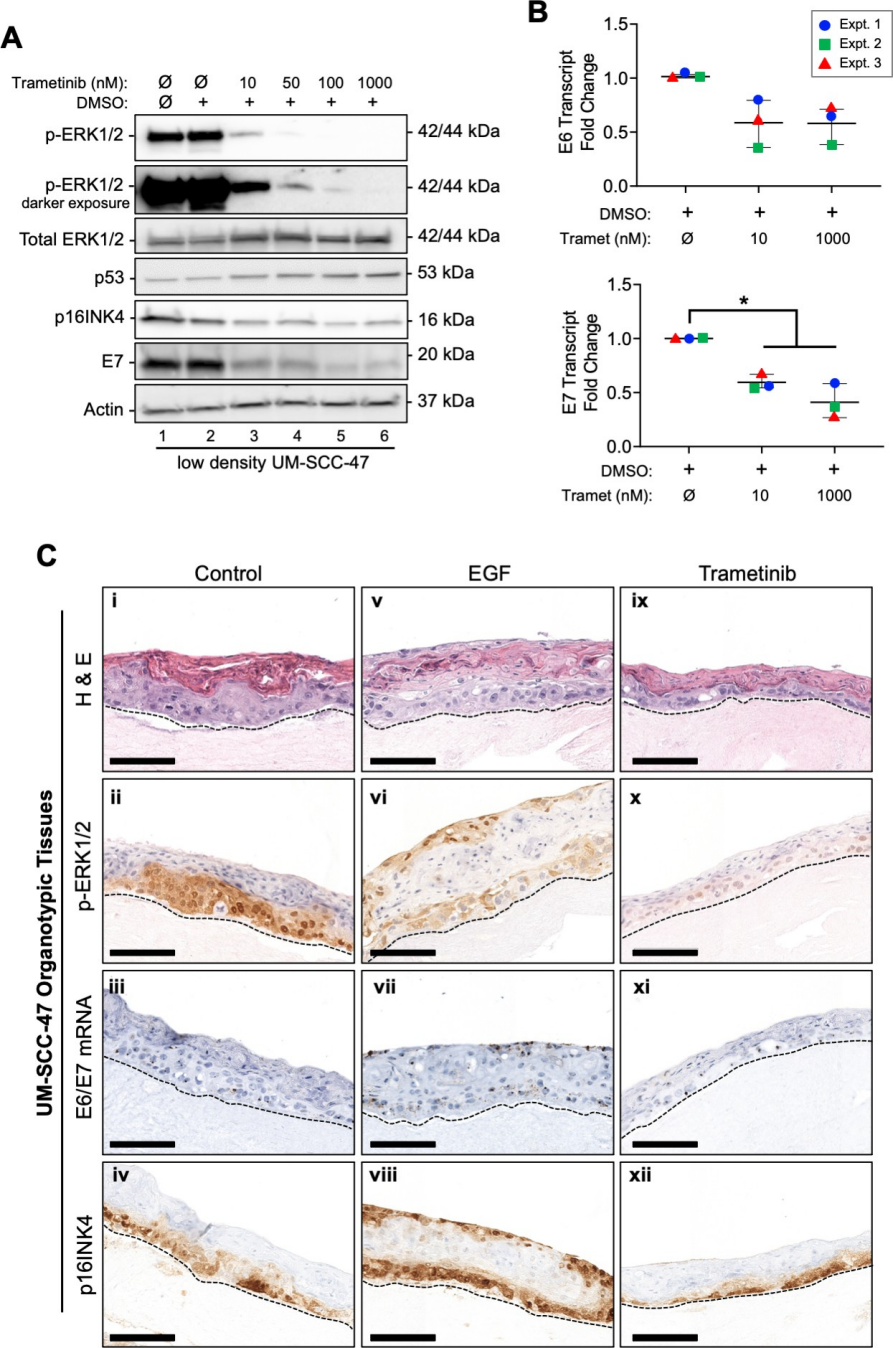
EGFR and MEK signaling regulates HPV oncogene expression in an HPV(+) OP-SCC cell line and 3D-organotypic epithelial tissues. (A-B) In parallel experiments, UM-SCC-47 cells were seeded at a low cell density for 16h before treating with increasing concentrations of trametinib or vehicle (0.1% DMSO) for 24h. (A) Protein lysates (25 μg) were analyzed by SDS-PAGE and IB. (B) Total RNAs extracted from replicate cultures subjected to RT-qPCR for HPV transcripts and normalized to ribosomal protein 18s mRNA levels. Vehicle treated (DMSO) values were set to one in each experiment and the data were analyzed by 1-way ANOVA with Dunnett’s T3 multiple comparison post hoc test (*p<0.05). (C) Organotypic cultures were grown for 8 days prior to treatment with 10 ng/ml EGF or 10 nM trametinib every second day. FFPE tissues harvested on day 14 were sectioned and stained: (i, v, ix) H&E; (ii, vi, x) IHC for p-ERK1/2; (iii, vii, xi) RNA ISH (brown chromogen); (iv, viii, xii) IHC for p16. Broken lines underscore the basal cell layer.

The experiments above demonstrated that HPV oncogene expression in the context of episomal genome replication is dependent upon EGFR/MEK/ERK signaling. However, many HPV-related human cancers harbor integrated viral genomes expressing E6 and E7. Although HPV genome integration disrupts the viral life cycle, the viral LCR invariably remains intact upstream of the viral early promoter that directs oncogene expression [64,65]. SiHa cervical SCC-derived cells containing integrated HPV16 genomes were shown to respond to EGF stimulation with enhanced E6/E7 mRNA expression dependent upon the viral LCR [32]. Thus, we hypothesized that other HPV(+) cancer cell lines would remain EGFR-signaling dependent. Rather than using cervical cancer cell lines with long and poorly defined culture history, we tested this hypothesis using the UM-SCC-47 cell line, which we obtained from the lab from which it was established. The UM-SCC-47 cell line was derived from the primary tumor of a moderately differentiated OP-SCC and harbors integrated HPV16 genomes [66]. The UM-SCC47 cell line was treated with increasing concentrations of trametinib for 24 hours. MEK inhibition resulted in dose-dependent suppression of p-ERK1/2, E7, and p16 protein expression (Fig 9A). As E6 protein expression was difficult to detect, we assayed p53 protein expression as a surrogate of E6 activity. We found that p53 levels increased concomitant with increasing trametinib concentrations, implying that E6 protein expression was decreased with MEK inhibition (Fig 9A). Furthermore, the reduction in HPV oncoprotein levels in response to MEK inhibition corresponded to decreased levels of E6 and E7 mRNAs (Fig 9B) similar to the outcomes in HPV(+) non-tumorigenic cell lines (Fig 5). Although p-ERK1/2 levels were nearly undetected at the highest doses of trametinib, E7 and p16 remained detectable. This suggests a mechanism in addition to MEK/ERK signaling may contribute to oncogene expression in these cells.

When UM-SCC-47 cells were cultured as organotypic epithelial tissues, the untreated tissues appeared partially differentiated, with a distinct uppermost cornified layer (Fig 9Ci). Nuclear p-ERK1/2, E6/E7 mRNAs, and p16 protein were confined to the middle and lower epithelial layers (Fig 9Cii–iv), suggesting an intact stratification program. However, EGF treatment led to epithelial proliferation resulting in overgrowth resembling hyperplasia and aberrant stratification; basal-like cells present at the apical tissue surface and an inverted cornified layer are suggestive of keratin pearls, a morphology consistent with SCC [67]. Likewise, p-ERK1/2, E6/E7 mRNAs, and p16 were increased in the non-cornified cells, including those at the apical surface (Fig 9Cv–viii). In contrast, trametinib-treated UM-SCC-47 epithelial tissues demonstrated a thickened apical cornified layer and greatly reduced p-ERK1/2 staining (Fig 9Cxi–x). Concurrently, the levels of E6/E7 transcripts and p16 protein staining were largely reduced (Fig 9Cxi–xii). Together with the findings in HPV(+) SiHa and UM-SCC-47 SCC cell monolayers, these findings demonstrate that the EGFR/MEK/ERK signaling pathway regulates HPV oncogene expression in the context of tissues expressing oncogenes from integrated HPV genomes.

## Discussion

**Fig 10:**
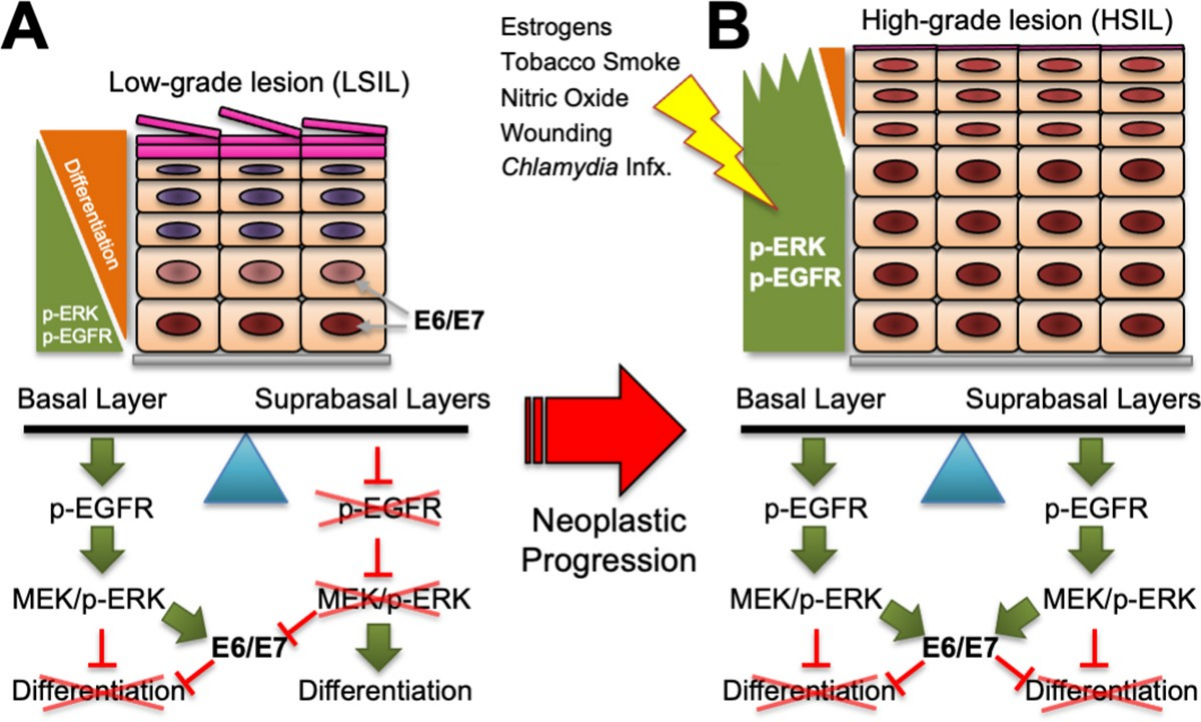
Model of the Proposed Mechanism of hr-HPV Oncogene Regulation by the MEK/ERK Signaling Pathway. A working model depicting the coordination of MEK/ERK signaling and hr-HPV E6/E7 oncoprotein expression leading to progressive loss of differentiation in early to late hr-HPV(+) neoplastic progression (adapted from [27]). (A) EGFR/MEK/ERK signaling in the basal layer is important for hr-HPV oncogene expression, which feeds back into enhancing ERK1/2 signaling and dampening terminal differentiation. In contrast, suppressed EGFR/MEK/ERK signaling in the suprabasal layers leads to reduced E6/E7 oncogene expression, permitting epithelial differentiation and expression of hr-HPV late proteins in LSIL. (B) Consistent with our data and reports that EGFR signaling and levels of p-ERK1/2 increase concomitant with HPV oncogene expression during progression from LSIL to HSIL, we find EGFR stimulation enhances E6/E7 oncogene expression. Our data predict that shifting the balance toward increased ERK1/2 signaling in suprabasal layers would cause increased E6/E7 oncogene expression and further loss of differentiation, fostering heightened neoplastic phenotypes in HSIL. Greater p-ERK1/2 signaling may result from cell-intrinsic alterations in upstream activators of ERK, or from cell-extrinsic factors often epidemiologically considered as risk-factors with hr-HPV infections progressing to HSIL and cancers. See the Discussion for specifics on how estrogen, tobacco smoke, nitric oxide, epithelial wounding and *Chlamydia trachomatis* infection (infx.) contribute to enhanced p-ERK1/2 signaling in the context of an hr-HPV infection and may serve to enhance cellular transformation and neoplastic progression.

Deregulated and maintained hr-HPV E6 and E7 oncogene expression is a central feature of HPV-induced pathogenesis and cancer progression. Numerous studies have shown that genetic suppression of oncogene expression results in cell cycle arrest, apoptosis, and tumor suppression [68–71]. Thus, these viral oncogenes are regarded as ideal therapeutic targets. Here, we aimed to understand the normal regulation of hr-HPV early gene expression in the context of epithelial infection, with a goal of revealing mechanisms underlying the loss of oncogene regulation that accompanies and is thought to promote neoplastic progression. Our results show that hr-HPVs rely on the EGFR/MEK/ERK signaling pathway to both activate and suppress oncogene expression depending upon the cellular and tissue context (Fig 10).

We investigated hr-HPV-infected cells and organotypic tissues that express viral genes from their native promoters in episomally-replicating viral genomes and show that hr-HPV oncogene transcription is regulated by contact inhibition, a cellular process in place to constrain proliferative signaling. Cellular contact inhibition is a mechanism that safeguards tissue homeostasis by uncoupling cell proliferation from mitogenic stimulation [72]. Loss of contact inhibition leads to hyperplasia *in vivo* and enables malignant progression by imparting a developing cancer cell with a propensity for unrestricted expansion [73]. Although the mechanisms controlling contact inhibition are incompletely defined, RTK signaling is regarded as an important regulator [48]. We focused on the role of EGFR/MEK/ERK signaling, as EGFR signaling is suppressed when cells become contact inhibited [48,50]. Specifically, we show that when cellular contact inhibition mechanisms suppress EGFR/MEK/ERK signaling, HPV oncogene transcription is concomitantly reduced. Furthermore, we find that EGFR ligand stimulation of high-density cells rescues ERK activation, temporarily overcomes contact inhibition, and promotes HPV oncogene expression. Thus, our data emphasize roles for EGFR and ERK signaling in both of these processes.

We also show in the context of contact inhibition that ERK signaling enhances the activity of the AP-1 TFs c-Fos, Fra-1, JunB and JunD without altering FosB or c-Jun activities. Nevertheless, knockdowns confirmed that c-Jun, as well as the p-ERK1/2-inducible activities of c-Fos and JunB contribute to oncogene transcription in the context of replicating HPV genomes, consistent with reductionist studies using LCR-driven reporter genes [35,36,60]. As active c-Jun levels remained relatively high in cells despite suppressed p-ERK1/2 signaling and oncogene expression, this implies that c-Jun heterodimers with c-Fos and/or JunB or c-Fos and JunB heterodimers may be involved in HPV oncogene transcription regulation under these conditions. Our data showing EGFR/ERK signaling to c-Fos, JunB and JunD activation are also consistent with reports that the expression of these AP-1 factors increases substantially in HPV(+) cervical cancers and cancer cell lines [38,39,62].

Our findings in organotypic epithelial tissue models and clinical cervical lesions provide a mechanistic link between the E6/E7 mRNA expression patterns observed in productive HPV(+) infections *in vivo* [9] with the well-characterized signaling patterns for EGFR and ERK in epithelia. EGFR plays a crucial role in normal epidermal development and physiology [74,75] with expression primarily localized to the undifferentiated basal and lower suprabasal layers [24,25]. EGFR expression is lost as cells exit the basal layers and this activity is accompanied by differentiation and loss of proliferation [26]. Patterns of p-ERK1/2 expression mirror those of EGFR in epithelia [76]. Herein we show the direct connection between the spatial expression of E6/E7 mRNAs (and the E7 surrogate, p16) and p-ERK1/2 signaling, consistent with the idea that EGFR/MEK/ERK signaling drives HPV oncogene expression in hr-HPV-infected epithelia (Fig 10A). Isaacson Wechsler et al. demonstrated positive correlations amongst increased hr-HPV E6 and E7 oncoprotein expression, the loss of contact inhibition, and more severe hyperplasia and neoplastic transformation [46]; our work herein corroborates and extends their observations. Specifically, they found that cells expressing higher E6 and E7 protein levels displayed decreased contact inhibition in monolayer cultures, and similarly produced more severe neoplastic phenotypes (HSIL-like) mirroring reduced contact inhibition in organotypic epithelial tissues. Additionally, they showed that cells expressing lower oncoprotein levels were more readily contact inhibited in monolayer and yielded mildly neoplastic (LSIL) phenotypes in 3D-tissue cultures. Our work supports and deepens mechanistic insight by showing that EGF stimulation concomitantly enhances both oncogene expression and the severity of the neoplastic phenotype in 3D-epithelial tissues. Together, these studies underscore the intimate link between oncogene expression and neoplastic progression, and our data further show that EGFR/MEK/ERK signaling is central to regulating these phenotypes.

Previous work indicates that hr-HPV oncoproteins, when ectopically over-expressed experimentally or in cancer-derived cells, augment EGFR signaling. For example, hr-HPV E6 and E7 each reportedly upregulate EGFR expression at the genomic level [77,78]. Hr-HPV E5 overexpression increases EGFR recycling to the cell surface after activation-induced EGFR internalization [79–82]. However, no prior studies have determined whether HPV oncoproteins influence EGFR signaling during an HPV infection when expressed in the context of replicating viral genomes. Given our finding that EGFR/MEK/ERK signaling promotes HPV oncogene expression, one might expect a progressive feed-forward loop to ensue in an HPV lesion whereby oncoproteins enhance EGFR signaling, which stimulates increased oncogene expression, and so on. However, our studies clearly show that the HPV(+) cells we studied, including non-transformed cells and the UNM-SCC-47 cancer cell line, remain responsive to the growth inhibitory cues of cell contact inhibition and epithelial tissue stratification/differentiation. These findings indicate that HPV oncoproteins do not dominate EGFR/MEK/ERK activities, but that EGFR/MEK/ERK signaling governs control over hr-HPV oncogene expression. These data underscore the importance of studying the functions of viral oncoproteins expressed from their natural promoters and within the context of other viral proteins.

In contrast to our findings, a few prior reports found some cell lines responded to EGFR stimulation with decreased E6/E7 transcription [32,43,83]. However, those studies relied on artificial promoter/enhancers and/or used cells with HPV genomic fragments that might impede important oncoprotein-cell signaling crosstalk and could account for the different outcomes. It is also possible that EGFR-independent ERK signaling pathways can influence HPV oncogene expression. Such situations may arise during the development of HPV(+) cancers that have mutations in *PIK3CA* (or downstream effectors) and/or become refractory to EGFR or PI3K inhibitors, which we and others reported can lead to MEK/ERK stimulation *via* other RTKs [84–87].

Herein, we show the direct correlation amongst clinical features of cervical neoplastic transformation, p-ERK1/2 levels, and the E7 surrogate, p16. This consolidates data from prior studies showing both increased EGFR and p-ERK1/2 signaling with HPV(+) CIN grade [29,31], and reports of paralleling increases in hr-HPV E6 and E7 mRNA levels during neoplastic progression [9]. These findings prompt us to speculate that dysregulation of EGFR and/or MEK/ERK signaling promotes increased hr-HPV oncogene expression during CIN lesion progression to cancer (Fig 10B). MEK/ERK signaling, whether induced by extrinsic ligand activation of EGFR other RTKs or intrinsic genetic mutations in upstream ERK activators may be a key driving force in neoplastic progression of hr-HPV(+) lesions by both promoting proliferation and enhancing oncogene expression. Our data suggest that hr-HPVs, and possibly all epithelial-tropic papillomaviruses, have evolved to exploit the MEK/ERK pathway to regulate their productive replicative cycles. As EGFR/MEK/ERK signaling decreases during normal differentiation, this both suppresses oncogene expression and promotes epithelial differentiation essential for late viral gene expression (Fig 10A). Yet, this MEK/ERK regulation can be a double-edged sword if MEK/ERK signaling is enhanced in HPV-infected tissues, potentially by either intrinsic or extrinsic means (Fig 9B). Thereby, increased proliferation prevents differentiation-induced late viral events, and increased dysplastic growth is promoted by both cellular- and viral oncoprotein-mediated mechanisms. As demonstrated by studies analyzing HPV(+) cervical and OP-SCCs [56,88], cell-intrinsic modes may include activating mutations in upstream ERK activators, including *EGFR/ERBB1*, *ERBB2*, *ERBB3*, *SRC*, *KRAS*, *NRAS*, *BRAF*. Extrinsic stimuli could include a wound response, or exposure to chemicals, pathogens, or other inflammatory agents that activate the MEK/ERK pathway in the epithelium. For example, we and other labs showed tobacco smoke components promote hr-HPV oncogene expression *via* MEK and/or AP-1 TF activation [89–91]. Nitric oxide, a free radical involved in cervical ripening during childbirth, activates ERK signaling [92]. We showed nitric oxide enhances HPV oncogene expression and survival of mutagenized cells, providing a potential mechanism by which multi-parity contributes to hr-HPV lesion progression [93]. Intracellular *Chlamydia trachomatis* infections promote MEK/ERK signaling [94,95], and we predict this could upregulate hr-HPV oncogene expression. Lastly, estrogen can activate ERK1/2 and thereby AP-1 TFs *via* the G protein-coupled receptor homolog, GPER, which both catalyzes the release of HB-EGF and signals through adenylyl cyclase (reviewed in [96]). Importantly, epidemiological studies often consider these extrinsic exposures as co-factors with oncogenic HPV infections promoting cancer progression [97], and, thus they may share the mechanism of ERK activation by which they contribute to cell proliferation and hr-HPV oncogene expression, persistence and/or disease severity.

Perhaps our most significant, yet somewhat unexpected, finding is that suppressing MEK/ERK signaling with pharmacological inhibitors has profound anti-viral effects on hr-HPV(+) cells. Further, the inhibitors effectively quash hr-HPV oncogene transcription and protein expression in both early neoplastic cells maintaining episomal genomes and in cancer cells with integrated HPV genomes. This is particularly noteworthy since genetic means of suppressing E6/E7 expression have shown these viral proteins are oncogenic drivers *in vitro* and preclinical mouse tumor models. Reduction of E6/E7 expression restores tumor suppressor functions, leads to apoptosis and/or senescence, and suppresses tumorigenicity in preclinical models [6,8,69,71,98]. However, genetic means to suppress HPV oncogenes are not yet particularly feasible in human studies. In contrast, many pharmacological inhibitors of MEK/ERK are currently approved by the U.S. FDA for use in a variety of cancers unrelated to HPV infections. Importantly, we show that targeting cellular pathways usurped by and conserved among HPVs is agnostic to hr-HPV genotypes, which is another major advantage over genetic approaches that must be tailored specifically for each HPV gene and genotype targeted. Thus, our work shows promise for clinical testing of clinically-approved inhibitors of MEK or ERK to treat HPV early neoplastic lesions and cancers. As the AP-1 TF binding sites are highly conserved across PV genomes, these inhibitors may well have anti-viral effects in other HPVs and animal PVs that typically only cause benign tumors.

In summary, intracellular pathogens, particularly those that replicate in the cell nucleus and/or rely on cellular proliferation, utilize host factors that are responsive to cellular signaling cues. Many of these pathogens have evolved to alter cell signals to enhance their replication and/or avoid immune recognition. Our work reveals that hr-HPV oncogene transcription from replicating viral genomes in monolayer cells and epithelial tissues is governed by MEK/ERK signaling. In addition, the control of oncogene expression by MEK/ERK signaling is maintained in at least a subset of cancer cells bearing integrated HPV genomes. These observations suggest that HPVs have adapted their replicative cycles to regulate oncogene expression in response to signaling pathways that guide the keratinocyte proliferation and differentiation. We suggest this provides an opportunity to use molecular inhibitors that target cellular and tissue factors to broadly impede pathogenic HPV infections. MEK or ERK inhibition in HPV-transformed cancers may offer the benefit of anti-viral effects by reducing viral oncoprotein activities and restoring tumor suppressor functions in addition to generally suppressing cellular proliferation; the former mechanisms may yield distinct advantages in treating HPV-positive cancers.

## Materials and methods

### Contact for reagent and resource sharing

Further information and requests for resources and reagents should be directed to by the lead author, Michelle Ozbun (mozbun@salud.unm.edu).

### Cell culture

The NIKS cell line (a gift from Paul Lambert, U. Wisc.-Madison) was derived from normal immortalized human foreskin keratinocytes [44]. NIKS-SG3 and the NIKS-1K cell lines (gifts from P. Lambert and John Doorbar, U. Cambridge, respectively) were created by transfection of circular, wild-type HPV16 genomes into NIKS cells, resulting in stable, episomally replicating viral genomes [45,46]. NIKS-SG3 cells maintain ≈50–100 copies of HPV16 per cell [45]; NIKS 1K cells maintain ≈200 copies of HPV16 per cell [46]. Similarly, NIKS-H18 cells were created by transfection of circular, wild-type HPV18 genomes into NIKS cells (a gift from John Doorbar). Cell lines W12-E (a gift from P. Lambert) and CIN-612 9E (a gift from Laimonis Laimins, Northwestern U.) were established from human cervical intraepithelial neoplasia grade 1 (CIN1) biopsies. W12-E cells (clone 20863) maintain episomal HPV16 genomes at ≈100 copies per cell [99,100]. CIN-612 9E cells maintain episomal HPV31 (subtype 31b) genomes at 500–100 copies per cell [101]. W12-E and CIN-612 9E cells were investigated at <30 passages after cloning. All keratinocyte cell lines were co-cultured with mitomycin C-treated J2-3T3 fibroblast feeder cells in E medium containing 10% fetal bovine serum (FBS; Atlanta Biologicals) and 10 ng/ml murine epidermal growth factor (EGF; Corning) as described previously [93,102]. J2-3T3 fibroblasts were propagated in high-glucose DMEM (Irvine Sci.) supplemented with 10% newborn calf serum (NCS, Hyclone), 2mM glutamine. Fibroblasts were treated with 24 μM mitomycin C (Sigma) for 2–4 h followed by washing 3x each with ≥ 5 ml of 1X phosphate buffered saline (PBS). The UM-SCC-47 cell line (a gift from Thomas Carey, U. Michigan), established from an OP-SCC of the lateral tongue [66], was grown in DMEM plus 10% FBS. Cell lines were authenticated by short tandem repeat analysis (IDEXX) and used within 15 passages of verification. For all experiments, keratinocytes were plated without feeder cells in E medium with 10% FBS; UM-SCC-47 cells were plated in DMEM with 10% FBS.

### Organotypic 3D-epithelial tissue cultures and immunohistochemistry (IHC)

Cells were expanded and grown as organotypic epithelial tissues at the air-medium interface with E medium plus 10% FBS as detailed [102,103]. Growing epithelial tissues were treated every 2^nd^ day with 20 mM 1,2-dioctanoyl-sn-glycerol (C8:0; Calbiochem). For some experiments, cultures were also treated with 10 ng/ml EGF, 0.1% dimethyl sulfoxide (DMSO; Thermo-Fisher), or 10 nM Trametinib in DMSO every 2^nd^ day beginning on day 8. After 14 days of growth, tissues were fixed in 10% neutral buffered formalin for 24h, room temperature, then embedded in paraffin (FFPE). Tissue sections (4 μm thick) were subject to hematoxylin and eosin (H&E) staining, RNA ISH or IHC. For IHC, antigens were unmasked by boiling in 10mM sodium citrate with 0.05% Tween-20 in a BioSB Tintoretriever pressure chamber for 15 min, 100°C, cooling to room temperature, and washing in PBS, then a 10-min H_2_O_2_ wash. For IHC detection of phospho (p)-ERK1/2, blocking was in 5% normal goat serum in TBS-T for 1 h at room temperature. Rabbit anti-p-p44/42 MAPK (T202/Y204) (Cell Signaling) was used at a 1:2000 dilution. Primary antibodies were applied in Signal Stain antibody diluent (Cell Signaling) and incubated overnight, 4°C. The sections were washed with TBS-T and subject to Signal Stain Boost Detection Reagent (Cell Signaling) and Signal Stain DAB (diaminobenzidine) Chromogen using the manufacturer’s recommendations. p16INK4A was detected using a mouse monoclonal antibody at 1:200 (Calbiochem) and performed by the UNM Cancer Center’s Human Tissue Repository Shared Resource. Organotypic tissue data were confirmed in independent experiments.

### Tumor Microarray (TMA) Immunohistochemical staining and scoring

Tissue specimens from 250 patients with cervical intraepithelial neoplasia (CIN) and 330 normal epithelium tissues were constructed into Tissue microarrays (TMAs) as previously described [29]. Paraffin blocks were provided by the Korea Gynecologic Cancer Bank through Bio & Medical Technology Development Program of the Ministry of Education, Science and Technology, Korea (NRF-2017M3A9B8069610). This study was approved by the Institutional Review Board of Gangnam Severance Hospital (Seoul, South Korea), and all procedures were conducted in accordance with the guidelines of the Declaration of Helsinki.

The TMA sections were deparaffinized, rehydrated, and subjected to heat-induced antigen retrieval using antigen retrieval buffer of pH 6.0 (for p-ERK1/2) or pH 9.0 (for p16INK4) (Dako, Carpinteria, CA). All slides were quenched for 10 min in 3% H_2_O_2_ to block for endogenous peroxidase. The sections were incubated with primary antibodies against p-ERK1/2 (Cell Signaling; clone #20G11; 1:200 dilution) and p16INK4 (BD Pharmingen; clone #G175-405; 1:1000 dilution) for 1 hr at room temperature. The antigen-antibody reaction was visualized with the Dako EnVision^+^ Dual Link System-HRP (Dako) and DAB^+^ (3, 3’-diaminobenzidine; Dako). The slides were lightly counterstained with hematoxylin and then reviewed by light microscopy. The control included immunoglobulin G (IgG) and omission of the primary antibody.

The stained TMA sections were digitized using the NanoZoomer 2.0 HT (Hamamatsu Photonics K.K., Japan) at ×20 objective magnification. Digital analysis of the images was performed using Visiopharm Integrator System v6.5.0.2303 (VIS; Visiopharm, Hørsholm, Denmark). The mean intensity of DAB for each defined image was measured for quantification, and was categorized as follows: 0 = negative, 1 = weak, 2 = moderate, and 3 = strong. The final immunostaining score was calculated by multiplying the staining intensity and percentage of positive cells (possible range 0–300). The TMA contains 253 cases of cervical intraepithelial neoplasia, however, due to the complexity of sectioning, staining, as well as the heterogeneity of the samples, only 249 and 224 samples could be interpreted for the p16INK4 and pERK1/2, respectively.

### Nucleic acid collection and analyses

RNA and DNA were extracted from cells with TriReagent (Sigma) per the manufacturer’s directions. RNA was DNase treated (TURBO DNA-free kit, Ambion). Reverse transcription (RT) of total RNAs (0.5 μg each) was performed at 42°C, 60 min. Viral and cellular transcripts were subject to quantitative polymerase chain reaction (qPCR) on cDNAs as previously reported [93]; qPCR primers are summarized in S1 Table and illustrated in S1 Fig. The HPV transcripts targeted included unspliced E6 and E7 ORFs (Bio-Rad SsoFast EvaGreen Supermix) and spliced E1^E4 mRNA that also contains the E5 ORF (E1^E4/E5) (Bio-Rad iQ Supermix) (see S1 Fig). PCR for cDNA of *FOS*, *JUN*, *JUNB* was performed with PrimePCR PreAmp for SYBR Green Assay (BioRad). RT-qPCR data for target mRNAs were normalized to ribosomal protein 18s mRNA levels (Bio-Rad iQ Supermix) performed on the same cDNAs. qPCR was performed on a Bio-Rad CFX96 and analyzed using Bio-Rad CFX Manager (version1.6.541.1068).

### RNA *in situ* hybridization (ISH)

Cells grown on chamber slides and FFPE raft tissue sections were fixed in 10% NBF. Cells and tissues were stained according to the manufacturer’s protocol (2.5 HD Detection Kit–Brown) using a probe to hr-HPV RNAs containing the E6/E7 open reading frames (HPV HR7 probe, Advanced Cell Diagnostics). Signal detection was performed with ACD DAB. Tissues were counterstained with 50% Gill’s hematoxylin solution number 1. The specificity of RNA ISH was determined by RNase treatment.

### Protein isolation and immunoblotting

Cells were lysed on ice in RIPA buffer (50 mM Tris, 150 mM NaCl, 1% Triton X-100, 0.1% SDS, 5 mM EDTA, 1% deoxycholic acid) supplemented with 1X HALT protease/phosphatase inhibitor (Pierce), and 0.2mM sodium orthovanadate. For detection of HPV16 E6, cells were extracted in 1X lysis buffer (43.9mM HEPES, pH 7.5; 131.7mM NaCl; 1.1% Triton X-100; 8.8% glycerol; 1x protease inhibitor cocktail; 1mM PMSF; 1mM EGTA). Samples were centrifuged at 12K x g for 10 min at 4°C and supernatants transferred to fresh tubes. Protein concentrations were determined by Bradford assay (Bio-Rad Protein Reagent). Sample loading buffer (6X) (62.5 mM Tris-HCl pH 6.8, 2% sodium dodecyl sulfate, 40% glycerol, 0.05% bromophenol blue) with 0.05% β-mercaptoethanol was added to samples (1X final concentration). Total proteins (typically 25–50 μg) were subjected to 4–12% sodium dodecyl sulfate-polyacrylamide gel electrophoresis (SDS-PAGE). Proteins were transferred to Immobilon-P PVDF membrane (Bio-Rad) using immunoblot transfer buffer (0.25 M Tris, 0.192 M glycine, 20% methanol). Membranes were blocked with bovine serum albumin in Tris-buffered saline-Tween-20 (TBS-T; 20 mM Tris, 137 mM NaCl, 0.1% Tween-20) and incubated with primary antibodies overnight at 4°C. Antibodies purchased from Cell Signaling: p-EGFR (Y845) and EGFR, p-p44/42 MAPK (Y292, Y204), p44/42 MAPK, p-AKT, AKT, c-Fos, c-Jun, JunB, p53, and p16INK4a were used according to manufacturer’s recommendations. HRP-conjugated anti-mouse and anti-rabbit secondary antibodies (Cell Signaling or GE Healthcare) were used at a 1:5000 dilution. Membranes were stripped using mild PVDF stripping buffer (399.6 μM glycine, 3.5 μM SDS, 1% Tween 20, pH 2.2) for 8 min at room temperature followed by extensive washing in TBS-T. Stripped blots were re-blocked as described above then re-probed for β-actin as a loading control. To detect HPV16 E6 protein, 300μg of total protein lysate was subjected to SDS-PAGE and transferred to Immobilon-P PVDF membrane at 400mA, 90 min. Membranes were blocked overnight, 4°C then probed using an E6 antibody (Arbor Vita) at 5 μg/ml for 1 h, room temperature. The membrane was washed and secondary goat anti-mouse IgG-HRP (Jackson ImmunoResearch). Blots were visualized on a Bio-Rad ChemiDoc station or Konica SRX-101A film processor (Diagnostic Imaging Inc.) and analyzed with Fiji {Schindelin,2012}). All IB results were confirmed in ≥3 independent experiments.

### Flow cytometry

Keratinocytes were seeded without fibroblast feeder cells in E medium plus 10% FBS (lacking EGF) and allowed to attach overnight. Cells were dissociated using 0.25% trypsin-EDTA (Sigma), 20 min. Trypsin was quenched with complete E medium and cells were pelleted and washed with 2 ml cold 1X PBS. Cells were resuspended in 100 μl of cold 1x PBS and incubated with 2 μg AlexaFluor-647 labeled anti-EGFR antibody R-1 (Santa Cruz Biotech.) for 30 min, 4°C with mixing. Antibody binding beads from Quantum Simply Cellular kit (Bangs Labs.) were labeled concurrently for each experiment. Cells and beads were each washed and resuspended according to the manufacturer’s directions. Fluorescence signal was detected using a BD LSR Fortessa flow cytometer; data were analyzed using FACS DIVA software.

### Kinase inhibitors

Erlotinib, Trametinib, SCH772984 and BYL719 (Selleck) were reconstituted in DMSO. Dose-response curves were generated for each inhibitor to determine effective concentrations in cell lines.

### AP-1 transcription factor activity

W12-E cells were seeded at 1.3 x10^5^ cells/cm^2^ in a 6-well plate and allowed to adhere overnight. The cells were pretreated with DMSO or Trametinib for 8h prior to stimulation with 10 ng/ml EGF. Nuclear extracts were obtained using the Nuclear Extract Kit (Active Motif) following the manufacturer’s protocol. Active AP-1 transcription factors levels were measured using 5 μg of nuclear extract and following manufacturer protocol for the TransAM AP-1 Family ELISA (Active Motif).

### Transfection-mediated siRNA knockdown

W12-E cells were seeded at 1.49x10^4^ cells/cm^2^ in a 12 well plate format and allowed to adhere overnight. The cells were starved for 4 h with Opti-MEM (Gibco) to improve transfection efficiencies. Cells were transfected in Opti-MEM with 25 nM of ON-Target siRNA pools targeting *FOS*, *JUN* or *JUNB* transcripts (Horizon Discovery) and 1 ul of DharmaFECT 1 Transfection Reagent (Horizon Discovery). After 24 h the cells were harvested for protein and RNA or the medium was changed to complete E medium containing 10% calf serum and the cells were incubated for an additional 24 h before harvesting.

### Statistical analyses

All statistical analyses for growth curves and RT-qPCR experiments were performed in GraphPad Prism 8.0.1. For each replicate the viral targets were normalized to ribosomal protein 18s mRNA per experiment. Analyses of protein fold changes over time (Fig 3C) employed the random effects linear regression model with random slope. Analyses of the relative RNA levels with increasing cell density or drug treatment were based on the parametric 1-way Welch’s ANOVA with Dunnett’s T3 multiple comparison test. Analyses of RT-qPCR data from EGF-treated or untreated cells were compared by Welch’s *t*-test. Probability (*p*) values of less than 0.05 were considered to be statistically significant.

TMA IHC data were analyzed using IBM SPSS statistics version 21 (IBM Corporation, Armonk, NY). IHC cut-off for high expression of p-ERK1/2 and p16INK4 was determined through the receiver operating characteristic (ROC) analysis. The statistical comparisons of the differences in the protein expressions in the different groups were performed using non-parametric statistics (Kruskal-Wallis/Mann–Whitney U). Chi-square test was used to perform statistical comparisons for categorical variables. Correlation between protein expression was determined by Pearson’s (χ^2^ = 212.658, *p* <0.001). Results with two-tailed *p* values less than 0.05 were considered statistically significant.

## Supporting information

S1 Fig Organization of HPV16 and HPV31 genomes indicating general structures of the polycistronic early transcripts. **Related to Figs 2–6 and 9, S2 and S1 Table.** The circular genome of ≈7900 base pairs is linearized at the late polyadenylation (polyA) signal to illustrate the regulatory long control region (LCR), open reading frames, and main early mRNAs (A-E). Nucleotide numbering is below the thin horizontal rule (based on HPV16 [GenBank accession number: K02718], HPV31 [GenBank accession number: J04353]. Bent arrows mark the major early and late promoters; circles denote EGFR-responsive AP-1 transcription factor binding sites (AP-1 BS) [33]. Early and late polyA sites are indicated. Shaded boxes illustrate ORFs located in all three reading frames for each viral genome aligned with the polycistronic early transcripts (A-E). Each of the primer pairs for qPCR is represented by a node and the products as lines connecting each primer node. Long vertical lines demark the boundaries of the ORFs and the E6* and E1^E4 introns are shaded. Note that when the amplification products span introns, only spliced RNAs are amplified as the PCR cycle profile does not amplify >200 bp (verified by gel electrophoresis). The specificity of the products and their sizes is given for each (LCR, E6, E7, E1^E4). (A-E) Thick black lines represent noncoding sequences and thin lines mark introns.
(TIFF)

S2 Fig Increasing cell density reduces global tyrosine phosphorylation, EGFR/ERK signaling and HPV oncogene expression. **Related to Fig 3.** (A-B) Plates of decreasing surface area (21cm^2^, 9.0cm^2^ and 4.6cm^2^) were seeded with 1.25x10^6^ NIKS-SG3 cells that were allowed to grow for 24h. (A) Crystal violet staining shows increasing cell density from left to right (wedge). (B) Protein lysates were collected 24h post seeding of HPV16(+) cells (NIKS-SG3, NIKS-1K, W12-E), HPV18(+) cells (NIKS-HPV18), and HPV31(+) cells (CIN-612 9E). Lysates were subjected to SDS-PAGE and IB for total phospho-tyrosine and actin. (C-D) Experimental details as in Fig 3D–3F and S2A Fig. Antibodies recognizing HPV31 E6 and E7 proteins are not available. (E) W12-E cells subject to experimental details as in Fig 2G and Fig 2H. (F) CIN-612 9E cells analyzed for HPV31 E7 mRNAs as described for HPV16 in Fig 1A with experimental details as in Fig 3G and 3H. Scatterplots represent the mean and range of the data from 3 independent experiments. Analysis using 1-way ANOVA with Dunnett’s T3 multiple comparison test (*p<0.05, **p<0.01 or n.s., not significant). (G) Flow cytometry quantification of the number of plasma membrane-resident EGFR proteins on each cell line in subconfluent states. Scatterplots represent the center tendency and variability of the data (n.s., no significant differences compared to NIKS cells; analyzed by 1-way ANOVA with Tukey’s *post hoc* test).
(TIFF)

S3 Fig PI3K pathway inhibition does not impact HPV16 oncoprotein restoration after EGF stimulation. Contact inhibited W12-E cells were grown in the presence of various doses of an inhibitor of p110α (10 nM, 100 nM, 1 μM or 10 μM BYL719) for 8h. Cells were stimulated for 14h with 10ng/ml of EGF before the harvesting of protein for SDS-PAGE and IB.
(TIFF)

S1 Table p-ERK1/2 and p16INK4 expression^a^ in human cervical intraepithelial neoplasia (related to Fig 1). (DOCX)

S2 Table Primers used in analysis of HPV transcription and genome copies. (DOCX)

## References

[1] de Villiers EM, Fauquet C, Broker TR, Bernard HU, zur Hausen H. Classification of papillomaviruses. Virology. 2004;324:17–27. 10.1016/j.virol.2004.03.033 15183049

[2] zur Hausen H. Papillomaviruses and cancer: from basic studies to clinical application. Nat Rev Cancer. 2002;2:342–50. 10.1038/nrc798 12044010

[3] de Martel C, Ferlay J, Franceschi S, Vignat J, Bray F, Forman D, et al Global burden of cancers attributable to infections in 2008: a review and synthetic analysis. Lancet Oncol. 2012;13(6):607–15. 10.1016/S1470-2045(12)70137-7 22575588

[4] Giuliano AR, Nyitray AG, Kreimer AR, Pierce Campbell CM, Goodman MT, Sudenga SL, et al EUROGEN 2014 roadmap: Differences in human papillomavirus infection natural history, transmission and human papillomavirus-related cancer incidence by gender and anatomic site of infection. Int J Cancer. 2015;136(12):2752–60. 10.1002/ijc.29082 25043222 PMC4297584

[5] zur Hausen H. The search for infectious causes of human cancers: where and why. Virology. 2009;392(1):1–10. 10.1016/j.virol.2009.06.001 19720205

[6] Goodwin E, DiMaio D. Repression of human papillomavirus oncogenes in HeLa cervical carcinoma cells causes the orderly reactivation of dormant tumor suppressor pathways. Proc Natl Acad Sci. 2000;97(23):12513–8. 10.1073/pnas.97.23.12513 11070078 PMC18795

[7] Lee CJ, Suh EJ, Kang HT, Im JS, Um SJ, Park JS, et al Induction of senescence-like state and suppression of telomerase activity through inhibition of HPV E6/E7 gene expression in cells immortalized by HPV16 DNA. Exp Cell Res. 2002;277(2):173–82. 10.1006/excr.2002.5554 12083799

[8] von Knebel Doeberitz M, Rittmüller C, zur Hausen H, Dürst M. Inhibition of tumorigenicity of cervical cancer cells in nude mice by HPV E6-E7 anti-sense RNA. Int J Cancer. 1992;51(5):831–4. 10.1002/ijc.2910510527 1319412

[9] Doorbar J. The papillomavirus life cycle. J Clin Virol. 2005;32S:S7–S15. 10.1016/j.jcv.2004.12.006 15753007

[10] Thomas JT, Hubert WG, Ruesch MN, Laimins LA. Human papillomavirus type 31 oncoproteins E6 and E7 are required for the maintenance of episomes during the viral life cycle in normal keratinocytes. Proc Natl Acad Sci. 1999;96:8449–54. 10.1073/pnas.96.15.8449 10411895 PMC17536

[11] Flores ER, Allen HBL, Lee D, Lambert PF. The human papillomavirus type 16 E7 oncogene is required for the productive stage of the viral life cycle. J Virol. 2000;74(14):6622–31. 10.1128/jvi.74.14.6622-6631.2000 10864676 PMC112172

[12] Middleton K, Peh W, Southern S, Griffin H, Sotlar K, Nakahara T, et al Organization of human papillomavirus productive cycle during neoplastic progression provides a basis for the selection of diagnostic markers. J Virol. 2003;77(19):10186–201. 10.1128/jvi.77.19.10186-10201.2003 12970404 PMC228472

[13] Ozbun MA, Meyers C. Human papillomavirus type 31b E1 and E2 transcript expression correlates with vegetative viral genome amplification. Virology. 1998;248:218–30. 10.1006/viro.1998.9285 9721231 PMC3600430

[14] Fehrmann F, Klumpp DJ, Laimins LA. Human papillomavirus type 31 E5 protein supports cell cycle progression and activates late viral functions upon epithelial differentiation. J Virol. 2003;77(5):2819–31. 10.1128/jvi.77.5.2819-2831.2003 12584305 PMC149771

[15] Graham SV. Keratinocyte Differentiation-Dependent Human Papillomavirus Gene Regulation. Viruses. 2017;9(9):245 10.3390/v9090245 PMC5618011. 28867768 PMC5618011

[16] Doorbar J, Quint W, Banks L, Bravo IG, Stoler M, Broker TR, et al The biology and life-cycle of human papillomaviruses. Vaccine. 2012;30 Suppl 5:F55–70. 10.1016/j.vaccine.2012.06.083 23199966

[17] Stoler MH, Rhodes CR, Whitbeck A, Wolinsky SM, Chow LT, Broker TR. Human papillomavirus type 16 and 18 gene expression in cervical neoplasias. Human Pathol. 1992;23(2):117–28. 10.1016/0046-8177(92)90232-r 1310950

[18] Dürst M, Glitz D, Schneider A, zur Hausen H. Human papillomavirus type 16 (HPV 16) gene expression and DNA replication in cervical neoplasia: analysis by in situ hybridization. Virology. 1992;189(1):132–40. 10.1016/0042-6822(92)90688-l 1318602

[19] Bechtold V, Beard P, Raj K. Human papillomavirus type 16 E2 protein has no effect on transcription from episomal viral DNA. J Virol. 2003;77(3):2021–8. 10.1128/jvi.77.3.2021-2028.2003 12525636 PMC140940

[20] Cook PW, Pittelkow MR, Shipley GD. Growth factor-independent proliferation of normal human neonatal keratinocytes: Production of autocrine- and paracrine-acting mitogenic factors. J Cell Physiol. 1991;146(2):277–89. 10.1002/jcp.1041460213 1999476

[21] Pittelkow MR, Cook PW, Shipley GD, Derynck R, Coffey RJ. Autonomous growth of human keratinocytes requires epidermal growth factor receptor occupancy. Cell Growth Differ. 1993;4(6):513–21. 8373735

[22] Wang X, Bolotin D, Chu DH, Polak L, Williams T, Fuchs E. AP-2alpha: a regulator of EGF receptor signaling and proliferation in skin epidermis. J Cell Biol. 2006;172(3):409–21. 10.1083/jcb.200510002 16449191 PMC2063650

[23] Jost M, Kari C, Rodeck U. The EGF receptor–an essential regulator of multiple epidermal functions. Eur J Dermatol. 2000;10(7):505–10. 11056418

[24] Groves RW, Allen MH, MacDonald DM. Abnormal expression of epidermal growth factor receptor in cutaneous epithelial tumours. J Cutan Pathol. 1992;19(1):66–72. 10.1111/j.1600-0560.1992.tb01561.x 1556270

[25] Nanney LB, Stoscheck CM, Magid M, King LE. Altered [125I]epidermal growth factor binding and receptor distribution in psoriasis. J Invest Dermatol. 1986;86(3):260–5. 10.1111/1523-1747.ep12285389 3018088

[26] Peus D, Hamacher L, Pittelkow MR. EGF-receptor tyrosine kinase inhibition induces keratinocyte growth arrest and terminal differentiation. J Invest Dermatol. 1997;109(6):751–6. 10.1111/1523-1747.ep12340759 9406816

[27] Getsios S, Simpson CL, Kojima S-i, Harmon R, Sheu LJ, Dusek RL, et al Desmoglein 1–dependent suppression of EGFR signaling promotes epidermal differentiation and morphogenesis. J Cell Biol. 2009;185(7):1243–58. 10.1083/jcb.200809044 19546243 PMC2712955

[28] Grandis JR, Melhem MF, Barnes EL, Tweardy DJ. Quantitative immunohistochemical analysis of transforming growth factor-α and epidermal growth factor receptor in patients with squamous cell carcinoma of the head and neck. Cancer. 1996;78(6):1284–92. 10.1002/(SICI)1097-0142(19960915)78:6&lt;1284::AID-CNCR17&gt;3.0.CO;2-X 8826952

[29] Cho H, Chung J-Y, Song K-H, Noh KH, Kim BW, Chung EJ, et al Apoptosis inhibitor-5 overexpression is associated with tumor progression and poor prognosis in patients with cervical cancer. BMC Cancer. 2014;14(1):545 10.1186/1471-2407-14-545 25070070 PMC4125689

[30] Branca M, Ciotti M, Santini D, Bonito LD, Benedetto A, Giorgi C, et al Activation of the ERK/MAP kinase pathway in cervical intraepithelial neoplasia is related to grade of the lesion but not to high-risk human papillomavirus, virus clearance, or prognosis in cervical cancer. Am J Clin Pathol. 2004;122(6):902–11. 10.1309/VQXF-T880-JXC7-QD2W 15539382

[31] Li Q, Tang Y, Cheng X, Ji J, Zhang J, Zhou X. EGFR protein expression and gene amplification in squamous intraepithelial lesions and squamous cell carcinomas of the cervix. Int J Clin Exp Pathol. 2014;7(2):733–41. 24551297 PMC3925921

[32] Peto M, Tolle-Ersu I, Kreysch HG, Klock G. Epidermal growth factor induction of human papillomavirus type 16 E6/E7 mRNA in tumor cells involves two AP-1 binding sites in the viral enhancer. J Gen Virol. 1995;76(8):1945–58. 10.1099/0022-1317-76-8-1945 7636475

[33] Hess J, Angel P, Schorpp-Kistner M. AP-1 subunits: quarrel and harmony among siblings. J Cell Sci. 2004;117(25):5965–73. 10.1242/jcs.01589 15564374

[34] Bernard H-U. Regulatory elements in the viral genome. Virology. 2013;445(1–2):197–204. 10.1016/j.virol.2013.04.035 23725692

[35] Cripe TP, Alderborn A, Anderson RD, Parkkinen S, Bergman P, Haugen TH, et al Transcriptional activation of the human papillomavirus-16 P97 promoter by an 88-nucleotide enhancer containing distinct cell-dependent and AP-1-responsive modules. New Biol 1990;2(5):450–63. 1963084

[36] Thierry F, Spyrou G, Yaniv M, Howley P. Two AP1 sites binding JunB are essential for human papillomavirus type 18 transcription in keratinocytes. J Virol. 1992;66(6):3740–8. 10.1128/JVI.66.6.3740-3748.1992 1316480 PMC241159

[37] Kyo S, Klumpp D, Inoue M, Kanaya T, Laimins L. Expression of AP1 during cellular differentiation determines human papillomavirus E6/E7 expression in stratified epithelial cells. J Gen Virol. 1997;78(2):401–11. 10.1099/0022-1317-78-2-401 9018063

[38] Mahata S, Bharti AC, Shukla S, Tyagi A, Husain SA, Das BC. Berberine modulates AP-1 activity to suppress HPV transcription and downstream signaling to induce growth arrest and apoptosis in cervical cancer cells. Mol Cancer. 2011;10(1):39 10.1186/1476-4598-10-39 21496227 PMC3098825

[39] Prusty BK, Das BC. Constitutive activation of transcription factor AP-1 in cervical cancer and suppression of human papillomavirus (HPV) transcription and AP-1 activity in HeLa cells by curcumin. Int J Cancer. 2005;113(6):951–60. 10.1002/ijc.20668 15514944

[40] Rösl F, Das BC, Lengert M, Geletneky K, zur Hausen H. Antioxidant-induced changes of the AP-1 transcription complex are paralleled by a selective suppression of human papillomavirus transcription. J Virol. 1997;71(1):362–70. 10.1128/JVI.71.1.362-370.1997 8985358 PMC191059

[41] Soto U, Das BC, Lengert M, Finzer P, zur Hausen H, Rösl F. Conversion of HPV 18 positive non-tumorigenic HeLa-fibroblast hybrids to invasive growth involves loss of TNF-alpha mediated repression of viral transcription and modification of the AP-1 transcription complex. Oncogene. 1999;18(21):3187–98. 10.1038/sj.onc.1202765 10359524

[42] Tam SW, Shay JW, Pagano M. Differential Expression and Cell Cycle Regulation of the Cyclin-dependent Kinase 4 Inhibitor p16^INK4^. Cancer Res. 1994;54:5816–20. 7954407

[43] Yasumoto S, Taniguchi A, Sohma K. Epidermal growth factor (EGF) elicits down-regulation of human papillomavirus type 16 (HPV-16) E6/E7 mRNA at the transcriptional level in an EGF-stimulated human keratinocyte cell line: functional role of EGF-responsive silencer in the HPV-16 long control region. J Virol. 1991;65(4):2000–9. 10.1128/JVI.65.4.2000-2009.1991 1848315 PMC240041

[44] Allen-Hoffmann BL, Schlosser SJ, Ivarie CAR, Sattler CA, Meisner LF, O'Connor SL. Normal growth and differentiation in a spontaneously immortalized near-diploid human keratinocyte cell line, NIKS. J Investi Dermatol. 2000;114:444–55. 10.1046/j.1523-1747.2000.00869.x 10692102

[45] Genther SM, Sterling S, Duensing S, Münger K, Sattler C, Lambert PF. Quantitative role of the human papillomavirus type 16 E5 gene during the productive stage of the viral life cycle. J Virol. 2003;77(5):2832–42. 10.1128/jvi.77.5.2832-2842.2003 12584306 PMC149772

[46] Isaacson Wechsler E, Wang Q, Roberts I, Pagliarulo E, Jackson D, Untersperger C, et al Reconstruction of human papillomavirus type 16-mediated early-stage neoplasia implicates E6/E7 deregulation and the loss of contact inhibition in neoplastic progression. J Virol. 2012;86(11):6358–64. 10.1128/JVI.07069-11 22457518 PMC3372204

[47] McClatchey AI, Yap AS. Contact inhibition (of proliferation) redux. Curr Opi Cell Biol. 2012;24(5):685–94. 10.1016/j.ceb.2012.06.009 22835462

[48] Curto M, Cole BK, Lallemand D, Liu C-H, McClatchey AI. Contact-dependent inhibition of EGFR signaling by Nf2/Merlin. J Cell Biol. 2007;177(5):893–903. 10.1083/jcb.200703010 17548515 PMC2064288

[49] Mierzejewski K, Rozengurt E. Density-dependent inhibition of fibroblast growth is overcome by pure mitogenic factors. Nature. 1977;269(5624):155–6. 10.1038/269155a0 302916

[50] Swat A, Dolado I, Rojas JM, Nebreda AR. Cell density-dependent inhibition of epidermal growth factor receptor signaling by p38alpha mitogen-activated protein kinase via Sprouty2 downregulation. Mol Cell Biol. 2009;29(12):3332–43. 10.1128/MCB.01955-08 19364817 PMC2698726

[51] Kim J-H, Asthagiri AR. Matrix stiffening sensitizes epithelial cells to EGF and enables the loss of contact inhibition of proliferation. J Cell Sci. 2011;124(Pt 8):1280–7. 10.1242/jcs.078394 PMC3065384. 21429934 PMC3065384

[52] Moyer JD, Barbacci EG, Iwata KK, Arnold L, Boman B, Cunningham A, et al Induction of apoptosis and cell cycle arrest by CP-358,774, an inhibitor of epidermal growth factor receptor tyrosine kinase. Cancer Res. 1997;57(21):4838–48. 9354447

[53] Gilmartin AG, Bleam MR, Groy A, Moss KG, Minthorn EA, Kulkarni SG, et al GSK1120212 (JTP-74057) Is an Inhibitor of MEK Activity and Activation with Favorable Pharmacokinetic Properties for Sustained In Vivo Pathway Inhibition. Clin Cancer Res. 2011;17(5):989–1000. 10.1158/1078-0432.CCR-10-2200 21245089

[54] Morris EJ, Jha S, Restaino CR, Dayananth P, Zhu H, Cooper A, et al Discovery of a novel ERK inhibitor with activity in models of acquired resistance to BRAF and MEK inhibitors. Cancer Discov. 2013;3(7):742–50. 10.1158/2159-8290.CD-13-0070 23614898

[55] Ma YY, Wei SJ, Lin YC, Lung JC, Chang TC, Whang-Peng J, et al PIK3CA as an oncogene in cervical cancer. Oncogene. 2000;19(23):2739–44. 10.1038/sj.onc.1203597 10851074

[56] The Cancer Genome Atlas Research Network. Integrated genomic and molecular characterization of cervical cancer. Nature. 2017;543:378–84. 10.1038/nature21386 28112728 PMC5354998

[57] The Cancer Genome Atlas Network. Comprehensive genomic characterization of head and neck squamous cell carcinomas. Nature. 2015;517(7536):576–82. 10.1038/nature14129 25631445 PMC4311405

[58] Adiseshaiah P, Li J, Vaz M, Kalvakolanu DV, Reddy SP. ERK signaling regulates tumor promoter induced c-Jun recruitment at the Fra-1 promoter. Biochem Biophys Res Commun. 2008;371(2):304–8. 10.1016/j.bbrc.2008.04.063 18435914 PMC2441865

[59] Deng Z, Sui G, Rosa PM, Zhao W. Radiation-Induced c-Jun Activation Depends on MEK1-ERK1/2 Signaling Pathway in Microglial Cells. PLoS One. 2012;7(5):e36739 10.1371/journal.pone.0036739 22606284 PMC3351464

[60] Kikuchi K, Taniguchi A, Yasumoto S. Induction of the HPV16 enhancer activity by Jun-B and c-Fos through cooperation of the promoter-proximal AP-1 site and the epithelial cell type—specific regulatory element in fibroblasts. Virus genes. 1996;13(1):45–52. Epub 2nd Ed. 10.1007/BF00576977 8938978

[61] Tyagi A, Vishnoi K, Kaur H, Srivastava Y, Roy BG, Das BC, et al Cervical cancer stem cells manifest radioresistance: Association with upregulated AP-1 activity. Scientific reports. 2017;7(1):4781–14. 10.1038/s41598-017-05162-x PMC5500478. 28684765 PMC5500478

[62] de Wilde J, De-Castro Arce J, Snijders PJF, Meijer CJLM, Rösl F, Steenbergen RDM. Alterations in AP-1 and AP-1 regulatory genes during HPV-induced carcinogenesis. Cellular Oncology. 2008;30(1):77–87. 10.1155/2008/279656 18219112 PMC4618566

[63] Evans MF, Peng Z, Clark KM, Adamson CS-C, Ma X-J, Wu X, et al HPV E6/E7 RNA in situ hybridization signal patterns as biomarkers of three-tier cervical intraepithelial neoplasia grade. PLoS One. 2014;9(3):e91142 10.1371/journal.pone.0091142 PMC3953338. 24625757 PMC3953338

[64] Stein AP, Swick AD, Smith MA, Blitzer GC, Yang RZ, Saha S, et al Xenograft assessment of predictive biomarkers for standard head and neck cancer therapies. Cancer Med. 2015;4(5):699–712. 10.1002/cam4.387 25619980 PMC4430263

[65] Akagi K, Li J, Broutian TR, Padilla-Nash H, Xiao W, Jiang B, et al Genome-wide analysis of HPV integration in human cancers reveals recurrent, focal genomic instability. Genome Res. 2014;24(2):185–99. 10.1101/gr.164806.113 24201445 PMC3912410

[66] Bradford CR, Zhu S, Ogawa H, Ogawa T, Ubell M, Narayan A, et al P53 mutation correlates with cisplatin sensitivity in head and neck squamous cell carcinoma lines. Head Neck. 2003;25(8):654–61. 10.1002/hed.10274 12884349

[67] Kalele K, Kulkarni N, Kathariya R. Oral Squamous Cell Carcinoma: Hematoxylin and Eosin Staining. J Clin Diag Res. 2015;9(9):ZJ01 10.7860/JCDR/2015/13644.6416 PMC4606365. 26501036 PMC4606365

[68] Sima N, Wang W, Kong D, Deng D, Xu Q, Zhou J, et al RNA interference against HPV16 E7 oncogene leads to viral E6 and E7 suppression in cervical cancer cells and apoptosis via upregulation of Rb and p53. Apoptosis. 2008;13(2):273–81. 10.1007/s10495-007-0163-8 18060502

[69] Hsu DS, Kornepati AV, Glover W, Kennedy EM, Cullen BR. Targeting HPV16 DNA using CRISPR/Cas inhibits anal cancer growth in vivo. Future Virol. 2018;13(7):475–82. 10.2217/fvl-2018-0010 30245733 PMC6136077

[70] Hu Z, Yu L, Zhu D, Ding W, Wang X, Zhang C, et al Disruption of HPV16-E7 by CRISPR/Cas system induces apoptosis and growth inhibition in HPV16 positive human cervical cancer cells. BioMed Res Int. 2014;2014(3):612823–9. 10.1155/2014/612823 PMC4127252. 25136604 PMC4127252

[71] Zhen S, Hua L, Takahashi Y, Narita S, Liu Y-H, Li Y. In vitro and in vivo growth suppression of human papillomavirus 16-positive cervical cancer cells by CRISPR/Cas9. Biochem Biophys Res Comm. 2014;450(4):1422–6. 10.1016/j.bbrc.2014.07.014 25044113

[72] Carter SB. Tissue homeostasis and the biological basis of cancer. Nature. 1968;220(5171):970–4. 10.1038/220970a0 5701853

[73] Abercrombie M. Contact Inhibition and Malignancy. Nature. 1979;281(5729):259–62. 10.1038/281259a0 551275

[74] Miettinen PJ, Berger JE, Meneses J, Phung Y, Pedersen RA, Werb Z, et al Epithelial immaturity and multiorgan failure in mice lacking epidermal growth factor receptor. Nature. 1995;376(6538):337–41. 10.1038/376337a0 7630400

[75] Murillas R, Larcher F, Conti CJ, Santos M, Ullrich A, Jorcano JL. Expression of a dominant negative mutant of epidermal growth factor receptor in the epidermis of transgenic mice elicits striking alterations in hair follicle development and skin structure. EMBO J. 1995;14(21):5216–23. PMC394631. 7489711 10.1002/j.1460-2075.1995.tb00206.x PMC394631

[76] Pasonen-Seppanen S, Karvinen S, Torronen K, Hyttinen J, Jokela T, Lammi MJ, et al EGF upregulates, whereas TGF-beta downregulates, the hyaluronan synthases has2 and has3 in organotypic keratinocyte cultures: Correlations with epidermal proliferation and differentiation. J Invest Dermatol. 2003;120(6):1038–44. 10.1046/j.1523-1747.2003.12249.x 12787132

[77] Akerman GS, Tolleson WH, Brown KL, Zyzak LL, Mourateva E, Engin TSW, et al Human Papillomavirus Type 16 E6 and E7 Cooperate to Increase Epidermal Growth Factor Receptor (EGFR) mRNA Levels, Overcoming Mechanisms by which Excessive EGFR Signaling Shortens the Life Span of Normal Human Keratinocytes. Cancer Res. 2001;61(9):3837–43. 11325860

[78] Hu G, Liu W, Mendelsohn J, Ellis LM, Radinsky R, Andreeff M, et al Expression of epidermal growth factor receptor and human papillomavirus E6/E7 proteins in cervical carcinoma cells. J Natl Cancer Inst. 1997;89(17):1243–6. 10.1093/jnci/89.17.1243 9293917

[79] Crusius K, Auvinen E, Steuer B, Gaissert H, Alonso A. The Human Papillomavirus Type 16 E5-Protein Modulates Ligand-Dependent Activation of the EGF Receptor Family in the Human Epithelial Cell Line HaCaT. Exp Cell Res. 1998;241(1):76–83. 10.1006/excr.1998.4024 9633515

[80] Crusius K, Rodriguez I, Alonso A. The human papillomavirus type 16 E5 protein modulates ERK1/2 and p38 MAP kinase activation by an EGFR-independent process in stressed human keratinocytes. Virus Genes. 2000;20(1):65–9. 10.1023/a:1008112207824 10766308

[81] Straight SW, Hinkle PM, Jewers RJ, McCance DJ. The E5 oncoprotein of human papillomavirus type 16 transforms fibroblasts and effects the downregulation of the epidermal growth factor receptor in keratinocytes. J Virol. 1993;67(8):4521–32. 10.1128/JVI.67.8.4521-4532.1993 8392596 PMC237836

[82] Wetherill LF, Holmes KK, Verow M, Müller M, Howell G, Harris M, et al High-risk human papillomavirus E5 oncoprotein displays channel-forming activity sensitive to small-molecule inhibitors. J Virol. 2012;86(9):5341–51. 10.1128/JVI.06243-11 PMC3347351. 22357280 PMC3347351

[83] Rosenberger S, Arce JD-C, Langbein L, Steenbergen RDM, Rösl F. Alternative splicing of human papillomavirus type-16 E6/E6* early mRNA is coupled to EGF signaling via Erk1/2 activation. Proc Natl Acad Sci. 2010;107(15):7006–11. 10.1073/pnas.1002620107 20351270 PMC2872467

[84] Jiang N, Wang D, Hu Z, Shin HJC, Qian G, Rahman MA, et al Combination of anti-HER3 antibody MM-121/SAR256212 and cetuximab inhibits tumor growth in preclinical models of head and neck squamous cell carcinoma. Molec Cancer Ther. 2014;13(7):1826–36. 10.1158/1535-7163.MCT-13-1093 PMC4090261. 24748655 PMC4090261

[85] Pollock NI, Wang L, Wallweber G, Gooding WE, Huang W, Chenna A, et al Increased Expression of HER2, HER3, and HER2:HER3 Heterodimers in HPV-Positive HNSCC Using a Novel Proximity-Based Assay: Implications for Targeted Therapies. Clin Cancer Res. 2015;21(20):4597–606. 10.1158/1078-0432.CCR-14-3338 26138066 PMC4609280

[86] Brand TM, Hartmann S, Bhola NE, Li H, Zeng Y, O’Keefe RA, et al Cross-talk Signaling between HER3 and HPV16 E6 and E7 Mediates Resistance to PI3K Inhibitors in Head and Neck Cancer. Cancer Res. 2018;78(9):2383–95. 10.1158/0008-5472.CAN-17-1672 29440171 PMC6537867

[87] Brand TM, Hartmann S, Bhola NE, Peyser ND, Li H, Zeng Y, et al Human Papillomavirus Regulates HER3 Expression in Head and Neck Cancer: Implications for Targeted HER3 Therapy in HPV(+) Patients. Clin Cancer Res. 2017;23(12):3072–83. 10.1158/1078-0432.CCR-16-2203 27986750 PMC5474133

[88] Stransky N, Egloff AM, Tward AD, Kostic AD, Cibulskis K, Sivachenko A, et al The mutational landscape of head and neck squamous cell carcinoma. Science. 2011;333(6046):1157–60. 10.1126/science.1208130 21798893 PMC3415217

[89] Wei L, Griego AM, Chu M, Ozbun MA. Tobacco exposure results in increased E6 and E7 oncogene expression, DNA damage and mutation rates in cells maintaining episomal human papillomavirus 16 genomes. Carcinogenesis. 2014;35(10):2373–81. 10.1093/carcin/bgu156 25064354 PMC4178472

[90] Alam S, Conway MJ, Chen H-S, Meyers C. The Cigarette Smoke Carcinogen Benzo[a]pyrene Enhances Human Papillomavirus Synthesis. J Virol. 2008;82(2):1053–8. 10.1128/JVI.01813-07 17989183 PMC2224590

[91] Muñoz JP, Carrillo—Beltrán D, Aedo-Aguilera V, Calaf GM, León O, Maldonado E, et al Tobacco exposure enhances human papillomavirus 16 oncogene expression via EGFR/PI3K/Akt/c-Jun signaling pathway in cervical cancer cells Front Microbiol. 2018;9:3022 10.3389/fmicb.2018.03022 30619121 PMC6304352

[92] Hsieh SC, Lai CS, Chang CH, Yen JH, Huang SW, Feng CH, et al Nitric oxide: Is it the culprit for the continued expansion of keloids? Eur J Pharmacol. 2019;854:282–8. Epub 2019/04/30. 10.1016/j.ejphar.2019.04.040 31034822

[93] Wei L, Gravitt PE, Song H, Maldonado A, Ozbun MA. Nitric oxide induces early viral transcription coincident with increased DNA damage and mutation rates in human papillomavirus infected cells. Cancer Res. 2009;69(11):4878–84. 10.1158/0008-5472.CAN-08-4695 19487298 PMC3820841

[94] Patel AL, Chen X, Wood ST, Stuart ES, Arcaro KF, Molina DP, et al Activation of epidermal growth factor receptor is required for Chlamydia trachomatis development. BMC Microbiol. 2014;14(1):277 10.1186/s12866-014-0277-4 PMC4269859. 25471819 PMC4269859

[95] Su H, McClarty G, Dong F, Hatch GM, Pan ZK, Zhong G. Activation of Raf/MEK/ERK/cPLA2 signaling pathway is essential for chlamydial acquisition of host glycerophospholipids. J Biol Chem. 2004;279(10):9409–16. 10.1074/jbc.M312008200 14676189

[96] Prossnitz ER, Arterburn JB, Smith HO, Oprea TI, Sklar LA, Hathaway HJ. Estrogen Signaling through the Transmembrane G Protein-Coupled Receptor GPR30. Ann Rev Physiol. 2008;70(1):165–90. 10.1146/annurev.physiol.70.113006.100518 18271749

[97] Muñoz N, Castellsagué X, de González AB, Ggissmann L. Chapter 1: HPV in the etiology of human cancer. Vaccine. 2006;24 Suppl 3:S3-1–10. 10.1016/j.vaccine.2006.05.115 16949995

[98] Zhou J, Li B, Peng C, Wang F, Fu Z, Zhou C, et al Inhibition of cervical cancer cell growth in vitro and in vivo by lentiviral-vector mediated shRNA targeting the common promoter of HPV16 E6 and E7 oncogenes. Antiviral Res. 2013;98(2):305–13. 10.1016/j.antiviral.2013.03.010 23523766

[99] Stanley MA, Browne HM, Appleby M, Minson AC. Properties of a non-tumorigenic human cervical keratinocyte cell line. Int J Cancer. 1989;43:672–6. 10.1002/ijc.2910430422 2467886

[100] Jeon S, Allen-Hoffmann BL, Lambert PF. Integration of human papillomavirus type 16 into the human genome correlates with a selective growth advantage of cells. J Virol. 1995;69(5):2989–97. 10.1128/JVI.69.5.2989-2997.1995 7707525 PMC188998

[101] Bedell MA, Hudson JB, Golub TR, Turyk ME, Hosken M, Wilbanks GD, et al Amplification of human papillomavirus genomes in vitro is dependent on epithelial differentiation. J Virol. 1991;65(5):2254–60. 10.1128/JVI.65.5.2254-2260.1991 1850010 PMC240574

[102] Ozbun MA, Patterson NA. Using Organotypic (Raft) Epithelial Tissue Cultures for the Biosynthesis and Isolation of Infectious Human Papillomaviruses Curr Prot Microbiol. 34: John Wiley & Sons, Inc.; 2014 p. 14B.3.1–B.3.8. 10.1002/9780471729259.mc14b03s34 PMC4221589 25082004

[103] Arnette C, Koetsier JL, Hoover P, Getsios S, Green KJ. In Vitro Model of the Epidermis: Connecting Protein Function to 3D Structure. Methods Enzymol. 2016;569:287–308. 10.1016/bs.mie.2015.07.015 PMC4870045. 26778564 PMC4870045

