# Supplementary figures and images for: MEK/ERK signaling is a critical regulator of high-risk human papillomavirus oncogene expression revealing therapeutic targets for HPV-induced tumors
Source: PLoS Pathog. 2021 Jan 22;17(1):e1009216. doi: 10.1371/journal.ppat.1009216 (PMC7857559; doi:10.1371/journal.ppat.1009216)

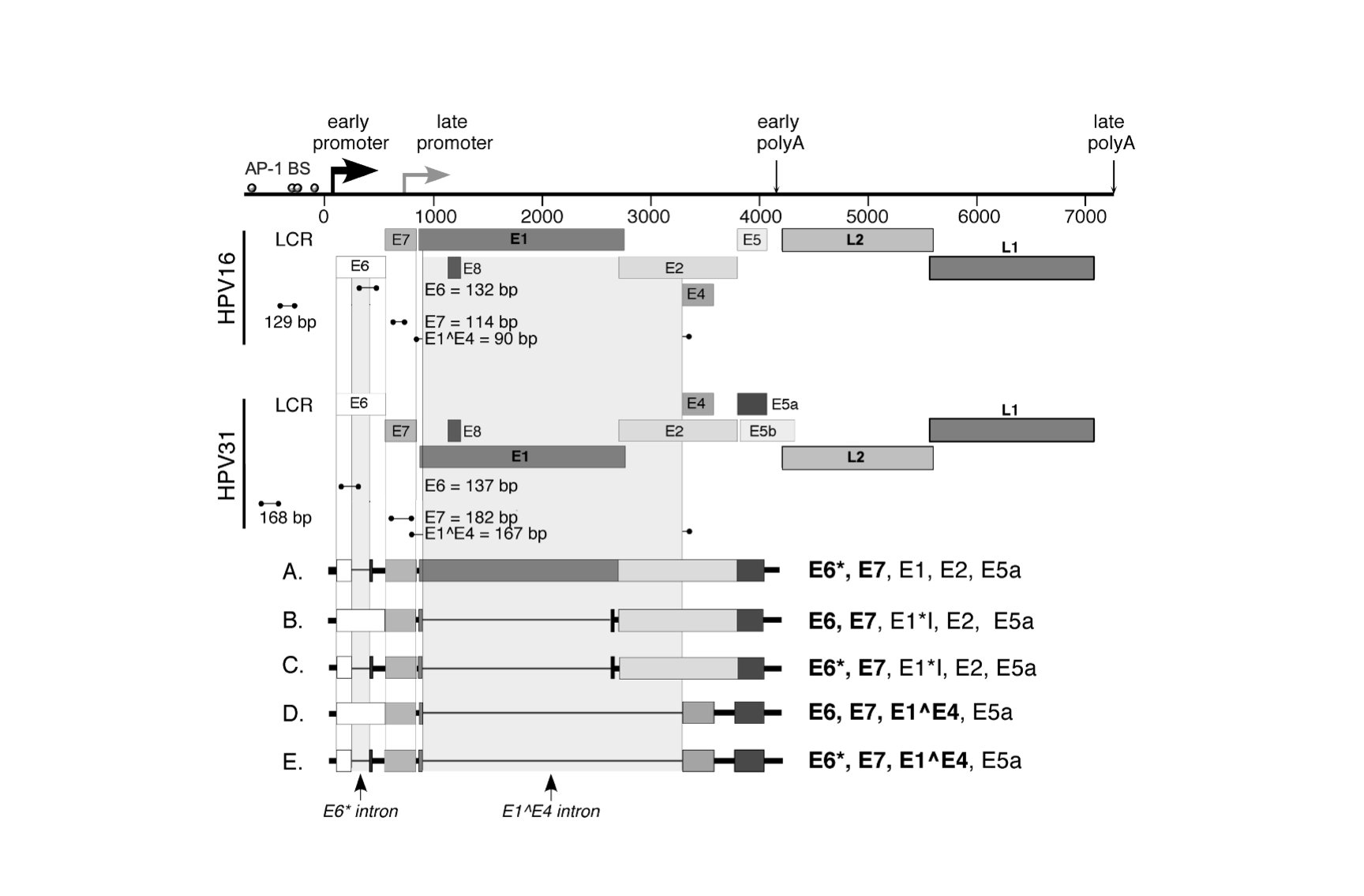

Supplement: S1 Fig — Related to Figs 2–6 and 9, S2 and S1 Table. The circular genome of ≈7900 base pairs is linearized at the late polyadenylation (polyA) signal to illustrate the regulatory long control region (LCR), open reading frames, and main early mRNAs (A-E). Nucleotide numbering is below the thin horizontal rule (based on HPV16 [GenBank accession number: K02718], HPV31 [GenBank accession number: J04353]. Bent arrows mark the major early and late promoters; circles denote EGFR-responsive AP-1 transcription factor binding sites (AP-1 BS) [33]. Early and late polyA sites are indicated. Shaded boxes illustrate ORFs located in all three reading frames for each viral genome aligned with the polycistronic early transcripts (A-E). Each of the primer pairs for qPCR is represented by a node and the products as lines connecting each primer node. Long vertical lines demark the boundaries of the ORFs and the E6* and E1^E4 introns are shaded. Note that when the amplification products span introns, only spliced RNAs are amplified as the PCR cycle profile does not amplify >200 bp (verified by gel electrophoresis). The specificity of the products and their sizes is given for each (LCR, E6, E7, E1^E4). (A-E) Thick black lines represent noncoding sequences and thin lines mark introns. (TIFF) [file ppat.1009216.s001.tiff]

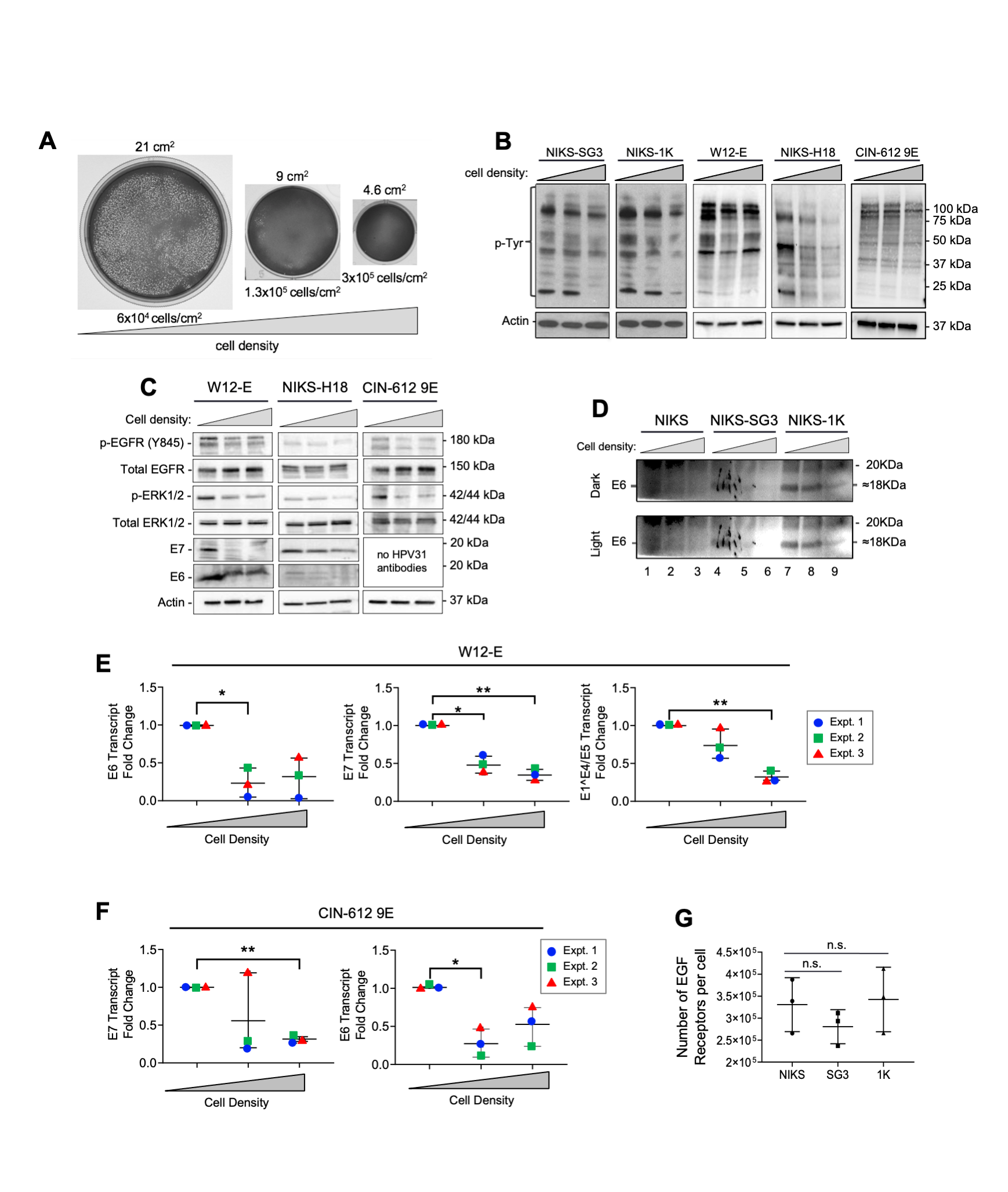

Supplement: S2 Fig — Related to Fig 3. (A-B) Plates of decreasing surface area (21cm2, 9.0cm2 and 4.6cm2) were seeded with 1.25x10^6^ NIKS-SG3 cells that were allowed to grow for 24h. (A) Crystal violet staining shows increasing cell density from left to right (wedge). (B) Protein lysates were collected 24h post seeding of HPV16(+) cells (NIKS-SG3, NIKS-1K, W12-E), HPV18(+) cells (NIKS-HPV18), and HPV31(+) cells (CIN-612 9E). Lysates were subjected to SDS-PAGE and IB for total phospho-tyrosine and actin. (C-D) Experimental details as in Fig 3D–3F and S2A Fig. Antibodies recognizing HPV31 E6 and E7 proteins are not available. (E) W12-E cells subject to experimental details as in Fig 2G and Fig 2H. (F) CIN-612 9E cells analyzed for HPV31 E7 mRNAs as described for HPV16 in Fig 1A with experimental details as in Fig 3G and 3H. Scatterplots represent the mean and range of the data from 3 independent experiments. Analysis using 1-way ANOVA with Dunnett’s T3 multiple comparison test (*p<0.05, **p<0.01 or n.s., not significant). (G) Flow cytometry quantification of the number of plasma membrane-resident EGFR proteins on each cell line in subconfluent states. Scatterplots represent the center tendency and variability of the data (n.s., no significant differences compared to NIKS cells; analyzed by 1-way ANOVA with Tukey’s post hoc test). (TIFF) [file ppat.1009216.s002.tiff]

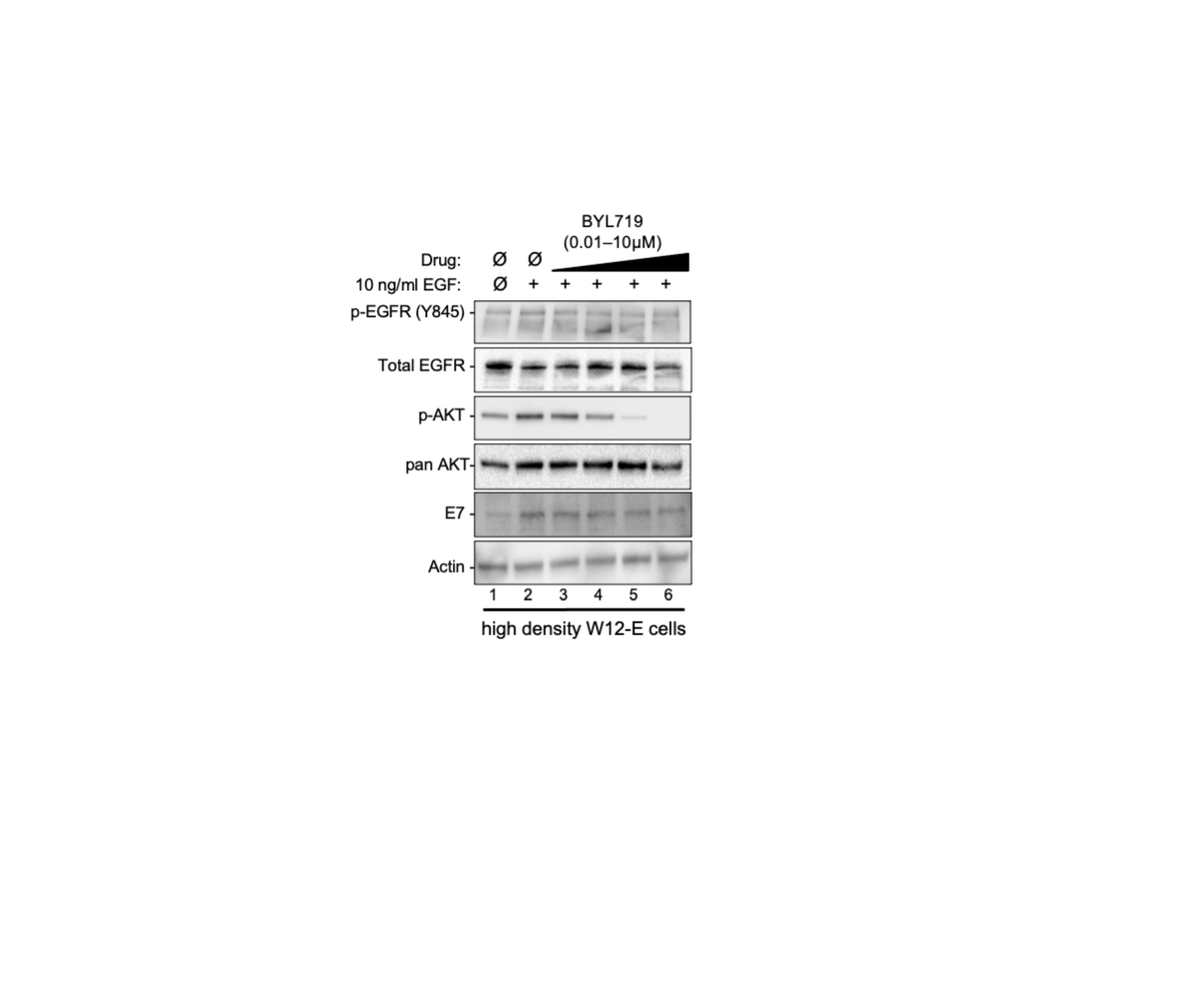

Supplement: S3 Fig — Contact inhibited W12-E cells were grown in the presence of various doses of an inhibitor of p110α (10 nM, 100 nM, 1 μM or 10 μM BYL719) for 8h. Cells were stimulated for 14h with 10ng/ml of EGF before the harvesting of protein for SDS-PAGE and IB. (TIFF) [file ppat.1009216.s003.tiff]
