# Supplementary material for: MEK/ERK signaling is a critical regulator of high-risk human papillomavirus oncogene expression revealing therapeutic targets for HPV-induced tumors
Source: PLoS Pathog. 2021 Jan 22;17(1):e1009216. doi: 10.1371/journal.ppat.1009216 (PMC7857559; doi:10.1371/journal.ppat.1009216)
Supplement: S1 Table — (DOCX) [file ppat.1009216.s004.docx]

**S1 Table. p-ERK1/2 and p16INK4 expression^a^ in human cervical intraepithelial neoplasia** **(related to Fig 1)**

|  | No. | p-ERK1/2 |  |  | No. | p16INK4 |  |
| --- | --- | --- | --- | --- | --- | --- | --- |
|  |  | Mean score (95% CI) | *p value^b^* |  |  | Mean score (95% CI) | *p value^b^* |
| **All study subjects** | 535 | 138.3 [132.8-143.8] |  |  | 577 | 99.0 [92.5-105.4] |  |
| **Diagnostic category** |  |  |  |  |  |  |  |
| Normal | 311 | 114.5 [108.1-121.0] |  |  | 328 | 52.9 [50.0-55.7] |  |
| Low-grade CIN | 49 | 147.3 [134.5-160.1] | *<0.001* |  | 69 | 94.7 [81.2-108.2] | *<0.001* |
| High-grade CIN | 175 | 177.9 [168.5-187.2] |  |  | 180 | 184.5 [172.9-196.1] |  |
| **HPV test in CIN** |  |  |  |  |  |  |  |
| Negative | 14 | 189.2 [156.8-221.5] | *0.201* |  | 20 | 138.4 [101.4-175.3] | *0.486* |
| Positive | 162 | 167.9 [158.7-177.2] |  |  | 180 | 151.7 [139.7-163.7] |  |

^a^ Protein expression was determined through analysis of an immunohistochemically stained tissue array, as described in the Materials and Methods section.

^b^ P-value was obtained from 1-way ANOVA when a variable has more than two categories and Welch’s *t*-test when it has two categories.

**S2 Table. Primers used in analysis of HPV transcription and genome copies.**

| **Primer** | **Sequence (5’ to 3’)^a^** | **Strand** | **Target mRNA** |
| --- | --- | --- | --- |
| **16QE6A** | GAACAGCAATACAACAACCG | Sense | HPV16 E6 |
| **16QE6B** | CCACCGACCCCTTATATTATG | Antisense | HPV16 E6 |
| **16QE7A** | CAGCTCAGAGGAGGAGGATG | Sense | HPV16 E7 |
| **16QE7B** | CACAACCGAAGCGTAGAGTC | Antisense | HPV16 E7 |
| **16QE1E4A** | CCATCTGTTCTCAGAAACCAT | Sense | HPV16 E1^E4 |
| **16QE1E4B** | GGCCAAGTGCTGCCTAAT | Antisense | HPV16 E1^E4 |
| **31QE6A** | GCTCGGCATTGGAAATACCC | Sense | HPV31 E6 |
| **31QE6B** | CTCCGTGTGGTGTGTCGTCC | Antisense | HPV31 E6 |
| **31QE7A** | ATGAGCAATTACCCGACAGC | Sense | HPV31 E7 |
| **31QE7B** | AGCCCATTAACAGCTCTTGC | Antisense | HPV31 E7 |
| **31QE1E4A** | GGCTCATTTGGAATCGTGTGC | Sense | HPV31 E1^E4 |
| **31QE1E4B** | CTTCACTGGTGCCCAAGG | Antisense | HPV31 E1^E4 |
| **16QE1E4 Probe** | ATACTTCGTTGCTGCTGCAGGATCAGCCAT |  | HPV16 E1^E4 |
| **31QE1E4 Probe** | CAGTGACGAAATATCCTTTGCTGGGATTGTT |  | HPV31 E1^E4 |
| **r18s** | Human RPS18 primer set (Bio-Rad, qHsaCEP0040177) |  | ribosomal protein 18s |

^a^ Corresponding to the sequence of HPV16 (GenBank accession number: K02718), HPV31 (GenBank accession number: J04353), or ß-actin (GenBank accession number: NM_001101.5)
