# Supplementary material for: MEK/ERK signaling is a critical regulator of high-risk human papillomavirus oncogene expression revealing therapeutic targets for HPV-induced tumors
Source: PLoS Pathog. 2021 Jan 22;17(1):e1009216. doi: 10.1371/journal.ppat.1009216 (PMC7857559; doi:10.1371/journal.ppat.1009216)
Supplement: S2 Table — (DOCX) [file ppat.1009216.s005.docx]

**S2 Table. Primers used in analysis of HPV transcription and genome copies.**

| **Primer** | **Sequence (5’ to 3’)^a^** | **Strand** | **Target mRNA** |
| --- | --- | --- | --- |
| **16QE6A** | GAACAGCAATACAACAACCG | Sense | HPV16 E6 |
| **16QE6B** | CCACCGACCCCTTATATTATG | Antisense | HPV16 E6 |
| **16QE7A** | CAGCTCAGAGGAGGAGGATG | Sense | HPV16 E7 |
| **16QE7B** | CACAACCGAAGCGTAGAGTC | Antisense | HPV16 E7 |
| **16QE1E4A** | CCATCTGTTCTCAGAAACCAT | Sense | HPV16 E1^E4 |
| **16QE1E4B** | GGCCAAGTGCTGCCTAAT | Antisense | HPV16 E1^E4 |
| **31QE6A** | GCTCGGCATTGGAAATACCC | Sense | HPV31 E6 |
| **31QE6B** | CTCCGTGTGGTGTGTCGTCC | Antisense | HPV31 E6 |
| **31QE7A** | ATGAGCAATTACCCGACAGC | Sense | HPV31 E7 |
| **31QE7B** | AGCCCATTAACAGCTCTTGC | Antisense | HPV31 E7 |
| **31QE1E4A** | GGCTCATTTGGAATCGTGTGC | Sense | HPV31 E1^E4 |
| **31QE1E4B** | CTTCACTGGTGCCCAAGG | Antisense | HPV31 E1^E4 |
| **16QE1E4 Probe** | ATACTTCGTTGCTGCTGCAGGATCAGCCAT |  | HPV16 E1^E4 |
| **31QE1E4 Probe** | CAGTGACGAAATATCCTTTGCTGGGATTGTT |  | HPV31 E1^E4 |
| **r18s** | Human RPS18 primer set (Bio-Rad, qHsaCEP0040177) |  | ribosomal protein 18s |

^a^ Corresponding to the sequence of HPV16 (GenBank accession number: K02718), HPV31 (GenBank accession number: J04353), or ß-actin (GenBank accession number: NM_001101.5)
